# Measuring affect dynamics: An empirical framework

**DOI:** 10.3758/s13428-022-01829-0

**Published:** 2022-04-05

**Authors:** Sergio Pirla, Maxime Taquet, Jordi Quoidbach

**Affiliations:** 1 Department of Economics and Business, Universitat Pompeu Fabra, Barcelona, Spain; 2 Department of Psychiatry, University of Oxford, Oxford, UK; 3 Oxford Health NHS Foundation Trust, Oxford, UK; 4 Universitat Ramon Llul, ESADE Business School, Barcelona, Spain

**Keywords:** Affect dynamics, Experience sampling method, Ambulatory assessment

## Abstract

A fast-growing body of evidence from experience sampling studies suggests that affect dynamics are associated with well-being and health. But heterogeneity in experience sampling approaches impedes reproducibility and scientific progress. Leveraging a large dataset of 7016 individuals, each providing over 50 affect reports, we introduce an empirically derived framework to help researchers design well-powered and efficient experience sampling studies. Our research reveals three general principles. First, a sample of 200 participants and 20 observations per person yields sufficient power to detect medium-sized associations for most affect dynamic measures. Second, for trait- and time-independent variability measures of affect (e.g., SD), distant sampling study designs (i.e., a few daily measurements spread out over several weeks) lead to more accurate estimates than close sampling study designs (i.e., many daily measurements concentrated over a few days), although differences in accuracy across sampling methods were inconsistent and of little practical significance for temporally dependent affect dynamic measures (i.e., RMSSD, autocorrelation coefficient, TKEO, and PAC). Third, across all affect dynamics measures, sampling exclusively on specific days or time windows leads to little to no improvement over sampling at random times. Because the ideal sampling approach varies for each affect dynamics measure, we provide a companion R package, an online calculator (https://sergiopirla.shinyapps.io/powerADapp), and a series of benchmark effect sizes to help researchers address three fundamental *hows* of experience sampling: *How many* participants to recruit? *How often* to solicit them? And for *how long*?

**Supplementary Information:**

The online version contains supplementary material available at 10.3758/s13428-022-01829-0.

## Introduction

With the advent of mobile phones, the *experience sampling method* (ESM; Csikszentmihalyi & Larson, 1984; also known as *ecological momentary assessment*; Stone & Shiffman, 1994) has quickly become a gold standard for studying human emotion (Lucas et al., 2021; Stone et al., 1998). Rather than relying on retrospective reports (“How did you feel yesterday?”) or cross-sectional surveys (“How do you feel in general?”), researchers in psychology, psychiatry, and behavioral science are now routinely capturing people’s subjective experience *in the moment* through short mobile questionnaires. Experience sampling not only alleviates recall and evaluative bias (Fredrickson & Kahneman, 1993; Redelmeier & Kahneman, 1996; Schimmack & Oishi, 2005), but also allows scientists to uncover how the dynamic aspects of people’s emotional lives (e.g., fluctuation, inertia) play a crucial role in mental and physical health (for a meta-analysis, see Houben et al., 2015).

Since the first ESM studies in the 1970s, countless articles have discussed the promise of the method for studying emotion (Ellison et al., 2020; Fisher & To, 2012; Myin-Germeys et al., 2018; Schimmack, 2003; Scollon et al., 2003), and many technical solutions have blossomed (see Arslan et al., 2019; Meers et al., 2020, for overviews). However, scientists have astonishingly been left to their own devices when it comes to *conducting* such research. Imagine, for example, that you want to assess how happy a person feels. How many moments of their daily life should you observe to capture their average happiness accurately? What about their propensity to experience mood swings? How spread in time or concentrated should your observations be? These questions are critical to the design of well-powered, cost-efficient ESM studies in affective sciences. However, an abysmal 2% of emotion ESM studies justify their sampling procedure (Trull & Ebner-Priemer, 2020), leading to important power, reproducibility, and suboptimal resource-allocation issues (e.g., Aguinis et al., 2013; Calamia, 2019; Kirtley et al. 2021).

In what follows, we first provide a brief overview of the experience sampling method in emotion research and the primary individual differences studied through this method. We then review the wide variety of sampling practices used to capture these individual differences. Finally, we stress the importance of relying on actual data to make critical decisions about how many participants to recruit and how often, when, and for how long to observe them.

### Experience sampling and affective sciences

Experience sampling involves repeated measurement of people's experience, as it unfolds in real time in their everyday lives (Conner et al., 2009). It offers several advantages over traditional lab- or survey-based emotion research.

First, by capturing emotions as they naturally occur in everyday life—rather than relying on artificial laboratory manipulation—ESM helps uncover how complex, intertwined, and diverse our affective reactions truly are (e.g., Dejonckheere et al., 2018; Kerr et al., 2020). For example, while theorists have debated the idea that people can experience two oppositely valenced emotions for decades, results from experience sampling suggest that this is a ubiquitous experience in everyday life: People report experiencing mixed emotions about a third of the time (Trampe et al., 2015).

Second, by capturing emotions in real time, ESM reduces recall and evaluative biases (e.g., Solhan et al., 2009; Stone et al., 1998). For example, people’s retrospective ratings of how they felt during emotional experiences are overly influenced by these experiences’ last and most intense moments (Fredrickson & Kahneman, 1993; Kahneman et al., 1993; Redelmeier & Kahneman, 1996). Similarly, global reports of affective states can be tainted by aspects of one’s life that happen to be salient at the moment (see Schimmack & Oishi, 2005, for a meta-analysis)—for example, asking people questions about politics right before asking them how happy they feel overall substantially reduces happiness scores (Deaton & Stone, 2016).

Third, by capturing emotions on multiple occasions, ESM allows us to study the influence of changing contexts on people’s emotions. For example, researchers have been able to quantify what type of daily activities (Choi et al., 2016; Taquet et al., 2016) or social interaction partners (Quoidbach et al., 2019) impact people’s momentary happiness. For instance, Mueller and colleagues (Mueller et al., 2019) examined over 50,000 episodes of social interactions. They found that social (vs. task-oriented) conversations with close (vs. less close) others were associated with higher momentary happiness.

### Experience sampling and affect dynamics measures

Beyond increased ecological validity and accuracy, a major contribution of ESM is that it allows researchers to uncover how individual differences in *affect dynamics—*that is, trajectories, patterns, and regularities in people’s emotion over time—play a critical role in mental health and psychopathology (Kuppens, 2015; Kuppens & Verduyn, 2017). Dozens of new affect dynamics measures have been introduced over the past decade, each designed to evaluate a unique aspect of people’s emotional lives. Whereas the incremental validity of several of these indicators is currently debated (Dejonckheere et al., 2019; Lapate & Heller, 2020; Wendt et al., 2020), the most common measures of affect dynamics in the literature include trait affect, affect variability, affect instability, and affect inertia (see Table 1).

**Table 1:** Affect dynamics measures included in our study. In the formulas, ${x}_{i}$ stands for the ${i}^{th}$ current affect report of a given individual. Similarly, n represents the total number of observations collected for the individual. *SD* and *M* represent respectively the standard deviation and mean affect reported by a given individual. Finally, $\mathrm{I}({\mathrm{X}}_{i+1}- {x}_{i}, {d}_{0.9})$ defines a binary variable taking a value of 1 if ${(x}_{i+1}- {x}_{i})$ is greater than ${d}_{0.9}$ in absolute terms and 0 otherwise, where ${d}_{0.9}$ represents the 90th percentile in the distribution of absolute affect changes across all participants in the sample

Measure	Index	Formal definition	Interpretation
Trait	Average (M)	$\frac{\sum {x}_{i}}{n}$	Average affect
Variability	Standard deviation (SD)	$\frac{\sum {{(x}_{i}-M)}^{2}}{n}$	Standard deviation of affect
Variability	Relative standard deviation (Rel. SD)	$\frac{SD}{max(SD|M)}$	Mean-corrected estimate of the standard deviation for bounded variables (Mestdagh et al., 2018)
Instability	Root mean square of successive differences (RMSSD)	$\sqrt{\frac{\sum {{(x}_{i}-{x}_{i+1})}^{2}}{n-1}}$	Average change across successive affect observations
Instability	Teager–Kaiser energy operator (TKEO)	$\frac{\sum(x_i^2-x_{i-1}\;\bullet\;x_{i+1})}{n-2}$	Measure of change across three affect reports. Useful in identifying mood spikes
Instability	Probability of acute change (PAC)	$\frac{\sum I( {x}_{i+1} - {x}_{i}, {d}_{0.9}) }{n-1}$	Likelihood of extreme affect changes
Inertia	Autocorrelation coefficient	$\frac{\sum ( {x}_{i} - M) ( {x}_{i+1} - M) }{\sum {( {x}_{i} - M)}^{2}}$	Correlation between successive affect reports

*Trait affect* represents people’s propensity to experience negative or positive affect and is considered a relatively stable personality characteristic (e.g., Watson & Tellegen, 1985). It is typically captured as the individual mean of affective states. *Affect variability* represents whether people’s affective state tends to change over time, regardless of when these changes occur. It is typically operationalized as the intra-individual standard deviation in affective states (Nesselroade & Salthouse, 2004; Ram & Gerstorf, 2009) or a mean-corrected version of this intra-individual standard deviation that avoids confounding effects of the mean (Mestdagh et al., 2018). In contrast, *affect instability* is a function of temporal order and represents whether people’s affective states tend to change abruptly from one moment to the next. Across different research domains, instability has been typically measured as the root mean square of successive differences (RMSSD; Jahng et al., 2008), the probability of acute change (PAC; Trull et al., 2008), or the Teager–Kaiser energy operator (TKEO; Solnik et al., 2010; Tsanas et al., 2016). Finally, *affect inertia* represents the degree to which people’s affective states persist from one moment to the next. It is typically captured as an autoregressive correlation between an individual’s current affective state and their previous affective state in time series (AR; e.g., Kuppens et al., 2010).

Accumulating empirical evidence shows that affect dynamics are associated with well-being and health. For example, research shows strong associations between average affect and depression (Golier et al., 2001; Thompson et al., 2012), post-traumatic stress disorder (Golier et al., 2001), borderline personality disorder (Zeigler-Hill & Abraham, 2006), and anxiety disorders (Bowen et al., 2006). Likewise, affect variability predicts lower subjective well-being (Gruber et al., 2013) and affective disorders (Bowen et al., 2004; Golier et al., 2001; McConville & Cooper, 1996). Affect instability is linked to poor mental health and several psychological disorders, including anxiety (Pfaltz et al., 2010), bipolar disorder (Jones et al., 2005), borderline personality disorder (Ebner-Priemer et al., 2007; Santangelo et al., 2014), major depressive disorder (Aan het Rot et al., 2012), and bulimia nervosa (Anestis et al., 2010). Finally, affect inertia is related to low self-esteem, neuroticism, and trait rumination (see Trull et al., 2015, for a review).

### Affect dynamics measures: the Wild West of sampling approaches

**Fig. 1:**
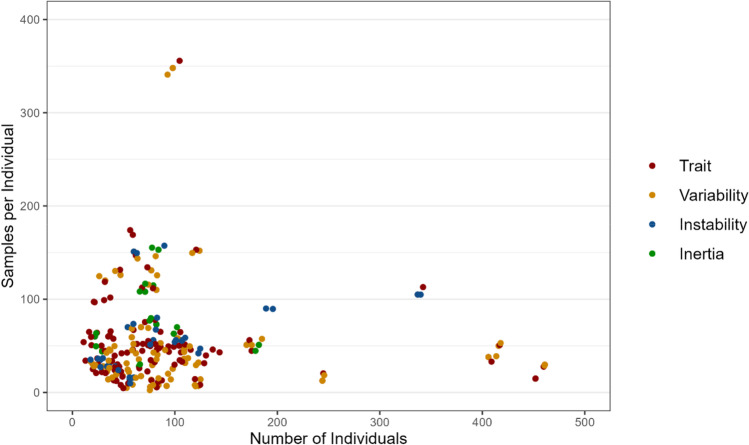
Distribution of the number of individuals sampled and the number of observations per individual in 88 emotion ESM studies

The field of affect dynamics holds great promise. But the wide range of outcomes that have been related to affect dynamics measures is met by an even wider range of methodological approaches to study them. We examined the sampling characteristics of 423 ambulatory assessment studies of affect included in five major review articles (Aan het Rot et al., 2012; Dunster et al., 2021; Ebner-Priemer & Trull, 2009; Houben et al., 2015; Myin-Germeys et al., 2009). Of these, 88 studies estimated at least one core affect dynamics measure. Our examination revealed a wide range of practices with samples ranging from 10 to 500 individuals and 14 to over 400 observations per individual (see Fig. 1). Studies also differed crucially with regard to when and for how long they surveyed participants. Some studies favored *close sampling*—many questionnaires collected over a short period (e.g., ten questionnaires a day for a week; Delespaul & DeVries, 1987; Myin-Germeys et al., 2000; Peeters et al., 2010)–whereas others favored *distant sampling*—few questionnaires per day collected over a longer period (e.g., two questionnaires a day for two weeks; Chepenik et al., 2006; Links et al., 2003). Some studies systematically sent questionnaires on *specific days* (weekdays vs. weekends; Beal & Ghandour, 2011) or at *specific times* (e.g., morning, afternoon, or evenings; Gruber et al., 2013; Knowles et al., 2007; Links et al., 2003; Zeigler-Hill & Abraham, 2006), while other studies probed participants at random times (Havermans et al., 2007; Peeters et al., 2006; Trull et al., 2008).

The lack of a standardized approach has profound ramifications. First, it leads researchers to rely on heuristics, opportunities, or unfounded conventions to define their sample size, rather than rely on adequate power calculation. For example, a common design in the ESM literature (around 40% of the studies) is to collect observations ten times a day for six consecutive days, even if this approach is neither based on power considerations nor necessarily optimal (Myin-Germeys et al., 2018). The current lack of evidence to guide sampling decisions might result in underpowered studies, leading to missed opportunities to discover true effects and inflated effect sizes of discovered effects (Ioannidis, 2008). Combined with publication bias and the difficulty to publish null results, underpowered studies are a root cause of the dire claim that most research findings are false (Ioannidis, 2005). Whereas underpowered studies are of great concern, researchers should not find solace in overpowered studies. Recruiting more participants than is needed or running a study for longer than necessary puts an unnecessary burden on participants, increases the risk of attrition, and misallocates essential resources. It might also be unethical if the answer to the research question at hand can improve people’s health or quality of life, and so should be sought with a degree of urgency.

### Developing an empirical framework

The goals of affective scientists when conducting experience sampling studies are twofold. First, they might be interested in precisely estimating an affect dynamic measure for a given group of individuals. Second, they might be interested in analyzing the relationship between an affect dynamic measure and another variable. In this paper, we consider both cases, presenting results that will be of use to those researchers concerned with estimation accuracy and those looking for guidance about power analysis.

A validated framework for study design would considerably advance the study of affect dynamics. But this framework needs to be determined on real affect data and not on simulations (Arend & Schäfer, 2019; Astivia et al., 2019; Lane & Hennes, 2018). In particular, while power analysis is a valid criterion to conduct inference under a set of plausible distributional assumptions of the data, defining a valid set of plausible distributional assumptions for affect dynamic studies is challenging. This is because the data generation process is complex and cannot be accurately captured by parametric models. Affect time series are stochastic processes that depend, in nonlinear ways, on various intertwined variables (e.g., time, weather, social interactions, cortisol level, physical wellness), many of which cannot be measured. Moreover, affect dynamics measures (e.g., the root mean squared successive differences) are themselves nonlinear summary statistics derived from these time series. Therefore, any valid framework to designing affect dynamics studies needs to link the probability distribution of these nonlinear transformations of non-uniformly sampled stochastic time series to the sampling process. In practice, this is most readily achieved using real data and assessing power empirically.

To address these issues, we build on a large dataset of 7016 individuals, each providing over 50 affect reports at random moments using smartphones. We first analyze how many samples are needed to accurately estimate a person’s affect dynamics in terms of trait affect (i.e., average), affect variability (i.e., within-person standard deviation), affect instability (i.e., RMSSD, TKEO, and PAC), and affect inertia (i.e., autocorrelation). We also investigate how strategic considerations in terms of timing between samples, time of the day, and days of the week change the number of samples needed to accurately estimate these affect dynamics measures. Second, we examine how the power to detect an association between the different measures and a given outcome varies as a function of sampling procedures. In doing so, we provide researchers with an easy-to-use companion R package and an online calculator to address the three fundamental *hows* of experience sampling studies: *How many* participants to recruit? *How often* to solicit them? And for *how long*?

## Method

### Participants and experience sampling

We collected our data using “58 s,” a free francophone smartphone application designed to assess different aspects of people’s well-being by sending short questionnaires at random times of the day. Participants provided basic information on age, gender, and country of residence at sign-up (see Note 1 of Supplementary Materials). They were then asked to select which days of the week, within what time windows, and how many sample requests they wanted to receive (default = 4 questionnaires daily between 9 a.m. and 10 p.m. each day of the week). Taking into account each user’s preferences and time constraints, the app sent questionnaire requests at random times throughout the day. By design, the minimum time between two consecutive notifications was set to 1 h. We ensured random sampling through a notification system that did not require users to be connected to the internet. Each questionnaire consisted of 4–6 questions selected from an extensive battery of items. The sample and item pool has been extensively described in other publications (Quoidbach et al., 2019; Taquet et al., 2020). For the purpose of this study, we focused on participants who reported their current affective state (using a slider from 0–*very unhappy* to 100–*very happy)* at least 50 times. This subsample included 7016 individuals (*M*_Age = _29.9, *SD*_Age_ = 9.9; 74% female) who each provided an average of 111.6 (*SD* = 87.8) momentary affect reports.

### Analytical approach

#### Estimating affect dynamics accurately

To analyze the number of reports required per individual to estimate each of the seven core affect dynamics measures reliably, we began by estimating their “true” value using the complete set of observations available for each individual. For example, if a participant provided 150 momentary affect reports, we computed the seven core affect dynamics measures for this participant (e.g., average happiness, within-person standard deviation, autoregressive coefficient) using all 150 observations. Then, we randomly selected a subset of *N* affect reports for each individual (with *N* varying from 3 to 30) and computed the affect dynamics measures using this smaller set of observations. We repeated this process 1000 times for each participant and for each value of *N*. We calculated an individual’s root mean square error (RMSE) of the estimates (compared to the “true” measure based on the full sample) for each value of *N*. We averaged the RMSE across participants to examine how the accuracy of the estimates changed as one increased the number of reports used to compute the different affect dynamics measures. To provide intuitive benchmarks against which these RMSE values can be compared, we also report, for each affect dynamics measure, the standard deviation of the “true” value in our population. This allows readers to appraise how big or small an RMSE is. For instance, if we were measuring people's weight, an RMSE of 1 g would be considered very small because the standard deviation of weights in the population is several kilograms. But if we were measuring insects' weights, an RMSE of 1 g would be considerably larger. If, for a given affect dynamics measure and number of affect reports per individual, our average RMSE equals one standard deviation in the true affect dynamics measure across individuals, we can expect the within-person estimation error to be equal in size to one between-person standard deviation in the true measure.

#### Optimizing sampling approaches

**Fig. 2:**
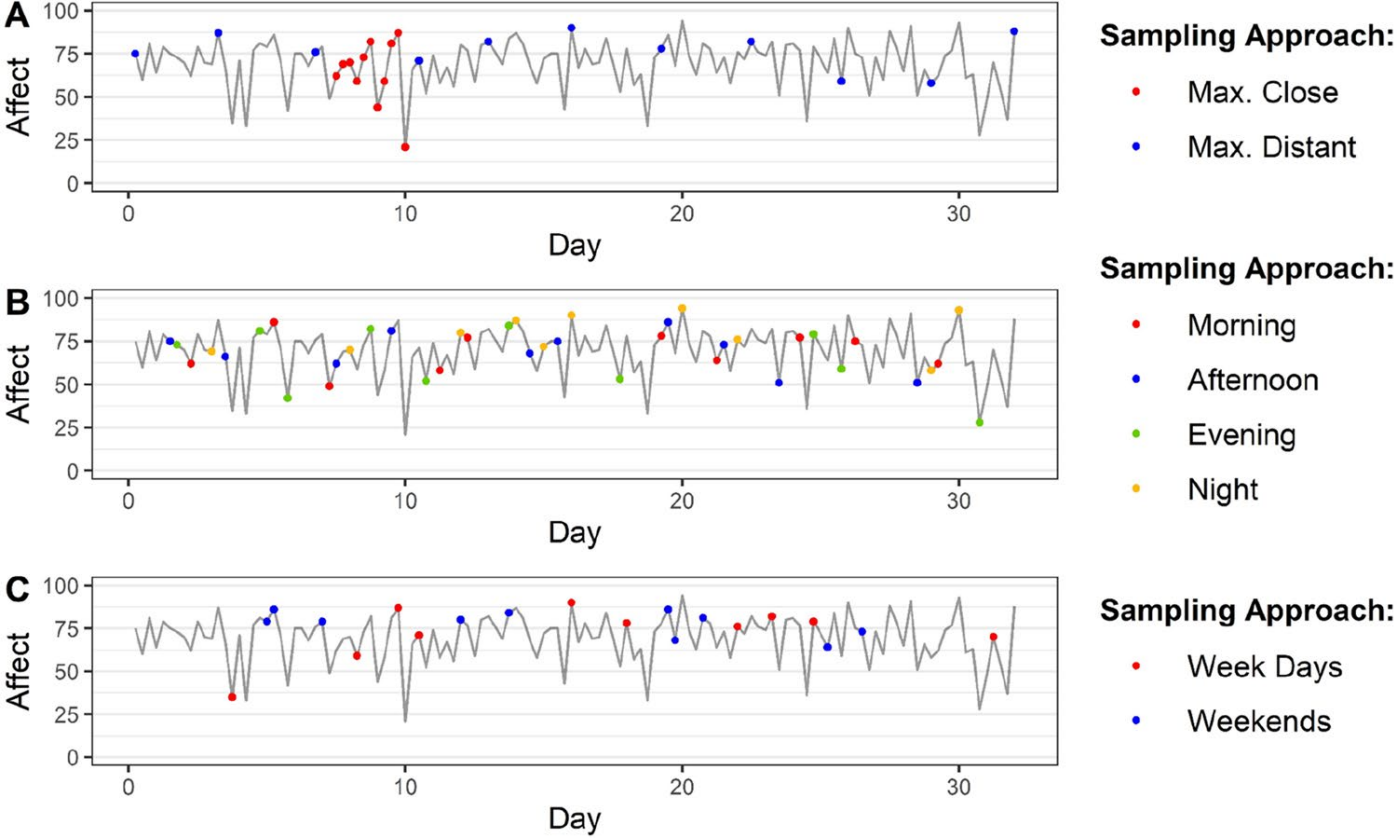
Graphical representation of the different sampling strategies tested

Could researchers reduce estimation errors of affect dynamics measures—and thus the number of reports required per individual—by probing participants at specific moments? To test whether sampling strategies can be optimized (see Fig. 2), we compared the accuracy of affect dynamics measures computed using reports selected at random times with affect dynamics measures computed with (1) temporally close or distant reports, (2) reports obtained at specific times, and (3) reports obtained on specific days (see details below).

To assess the accuracy of affect dynamics measures estimated using reports elicited at random times, we followed the procedure outlined in the previous section (see “Estimating affect dynamics accurately” section). These baseline accuracy estimates were then compared to those obtained using alternative sampling strategies. To assess our results’ robustness, for each condition and number of reports used, we bootstrapped over the individual-specific RMSE estimates to obtain the 95% confidence intervals for the average RMSE across individuals.

#### Random, close or distant sampling

Close sampling consists in collecting many reports over a short period of time. In this study, we consider close sampling to be the set of consecutive affect reports that were collected within the shortest possible time period for each individual (imposing a maximum of 24 h between each affect report). In contrast, distant sampling consists in collecting reports less frequently but for a longer period of time. In this study, we consider distant sampling to be the individual’s maximally distant reports. To determine an individual’s maximally distant reports, we divided the temporal window in which each participant provided reports (from their first to their last) into *N* − 1 equally spaced time intervals (where *N* takes on values between 3 and 30, depending on the number of reports used in the computation). We then computed the different affect dynamics measures, selecting reports that fell as close as possible to an equally spaced design. Note that by construction there is only a single set of reports for each individual that is considered close and distant sampling. Thus, for these sampling strategies, only one value of each affect dynamics measure was calculated per individual for each value of *N* (instead of resampling and estimating them 1000 times).

#### Random versus specific times sampling

Specific times sampling differs from random sampling in that we estimated the affect dynamics measures using reports collected exclusively in the morning (from 6 a.m. to 12 p.m.), afternoon (12 p.m. to 4 p.m.), evening (4 p.m. to 8 p.m.), or at night (8 p.m. to 6 a.m.). For each of these conditions and number of affect reports from 3 to 30, we resampled and estimated the affect dynamics measures 1000 times. We introduced a bias-correction term in the estimates of affect dynamics measures to account for any baseline differences that might exist between specific sampling times (e.g., on average, affect tends to be more pleasant in the evening than in the morning). To debias the estimates, we first estimated a time window-specific bias by subtracting from the population average of affect dynamics measures based on all available affect records, the population average of the same measure estimated with affect reports from our time window of interest. We then subtracted this bias from each of our estimates of affect dynamics measures averaged over 1000 bootstrap samples. For example, when analyzing the performance of the estimations of the average affect with reports collected at night, we first obtained a time window-specific bias. To calculate this bias term, we (1) estimated each individual’s average affect using all reports available, (2) estimated each individual’s average affect using all reports collected at night, (3) subtracted the population average of estimates in (1) from the population average of estimates in (2). The bias term is then added to each individual’s average affect. This debiasing procedure allowed us to account for “time-window fixed effects,” any bias across individuals that did not affect the relative ordering of individuals in terms of their affect dynamics measure of interest. Results obtained when excluding this bias-correction term can be found in Supplementary Note 2. For each time window, we excluded from our estimations participants that had not provided a minimum of 30 affect reports within that time window. This resulted in a final sample of 2806 individuals in the morning condition (i.e., 40% of the total sample), 2126 in the afternoon condition (i.e., 30.3% of the total sample), 2475 in the evening condition (i.e., 35.3% of the total sample), and 914 individuals in the night condition (i.e., 13% of the total sample).

#### Random versus specific days sampling

Specific days sampling differs from random sampling in that we estimated the affect dynamics measures using reports collected exclusively during the weekends (weekend sampling) or during the week (weekday sampling). For each of these conditions, we resampled and estimated each affect dynamics measure 1000 times using a specific number of reports from 3 to 30. Again, we included a bias-correction procedure and omitted the data from participants that did not provide a minimum of 30 affect reports in each condition. This resulted in a final sample of 6982 individuals in the weekday condition (i.e., 40% of the total sample), and 2482 individuals in the weekend condition (i.e., 13% of the total sample).

#### Statistical power as a function of sampling

In this section, we derive statistical power estimates for a two-tailed *t*-test on the Pearson correlation coefficient between a given variable and an affect dynamic measure. That is, given two variables (one of them being an affect dynamic measure), we analyze power for a two-tailed *t*-test examining the null hypothesis that the Pearson correlation between them is equal to zero, against the alternative hypothesis of a nonzero Pearson correlation coefficient. Throughout this paper, our tests employ a 0.05 significance level, but extensions of our analyses to different significance levels are included in our online calculator and R package.

To conduct these analyses, we first estimated the seven affect dynamics measures for each individual using all the observations at our disposal. We then simulated random variables displaying a weak (Pearson’s *r* = 0.10), medium (*r* = 0.30), and strong (*r* = 0.50) positive correlation with each affect dynamics measure by adding orthogonal random Gaussian noise (with a mean of 0 and standard deviation of 1) to projections of our variables of interest on vectors displaying the desired correlations. In doing so, we obtained variables displaying a weak, medium, and strong correlation with the affect dynamics measures derived from our full sample. We repeated this process to obtain a large enough set of simulated variables (2500 simulated variables per effect size and affect dynamic measure). To evaluate how the power to detect these correlations changes when affect dynamics measures are computed from smaller numbers of participants and smaller number of observations per participant, we considered ten different numbers of participants (*N*_Participants_ = 10, 20, 40, 80, 160, 320, 640, 1280, 2560, and 5120) and ten different numbers of observations per participants (*N*_Observations_ = 5, 10, 15, 20, 25, 30, 35, 40, 45, and 50), leading to 100 (= 10 × 10) sampling specifications in total.

For each combination of number of participants and number of observations per participant, we created 2500 datasets by resampling from our original data. For each of these 2500 datasets, we computed the seven affect dynamics measures for each participant. For each of these measures, we analyzed its correlation with a corresponding simulated variable (i.e., a simulated variable displaying the desired correlation with the full sample measure). We quantified power as the proportion of simulated datasets with a statistically significant positive correlation between the affect dynamics measures and the simulated variable.

#### Benchmarks for plausible effect sizes

Like other power calculation tools, the sampling recommendations derived from our empirical framework require researchers to anticipate plausible effect sizes for the association they are interested in (or to set a minimum effect size that they want their study to detect). Such anticipated effect sizes can be informed by systematic literature review, preliminary data, and meta-analyses. But in practice, it may be challenging for affective scientists to come up with realistic effect size estimates as the field of affect dynamics is relatively new, and such estimates may not exist. Moreover, historical data may offer little guidance as past estimates tend to be overestimates given reporting and publication bias favoring significant results (Gelman & Carlin, 2014). Therefore, we provide a series of benchmarks based on ten variables that we measured alongside affect in our experience sampling project: (1) age, (2) gender, (3) average sleep time, (4) life satisfaction, (5) meaning in life, as well as the proportion of time spent with (6) friends, (7) family, (8) alone, (9) working, and (10) exercising (see Supplementary Note 5 for the complete list of variables and their operationalization). Note that for life satisfaction and meaning in life, the associations we report are based on matched measures. For instance, we report the correlation between trait affect and trait life satisfaction, the correlation between affect instability and life satisfaction instability, and the correlation between affect inertia and life satisfaction inertia (vs. nonmatching pairs).

We chose to report these ten variables because they are commonly used demographic, well-being, and contextual measures in the experience sampling literature and cover a wide range of effect sizes—displaying correlations from |*r*|= 0.002 to |*r*|= 0.856 with our affect dynamic measures. By considering the magnitude of the relationships between these ten variables and the different affect dynamics measures, we hope to help researchers design optimized ESM studies based on plausible effect size estimates.

## Results

### Measuring affect dynamics accurately

**Fig. 3:**
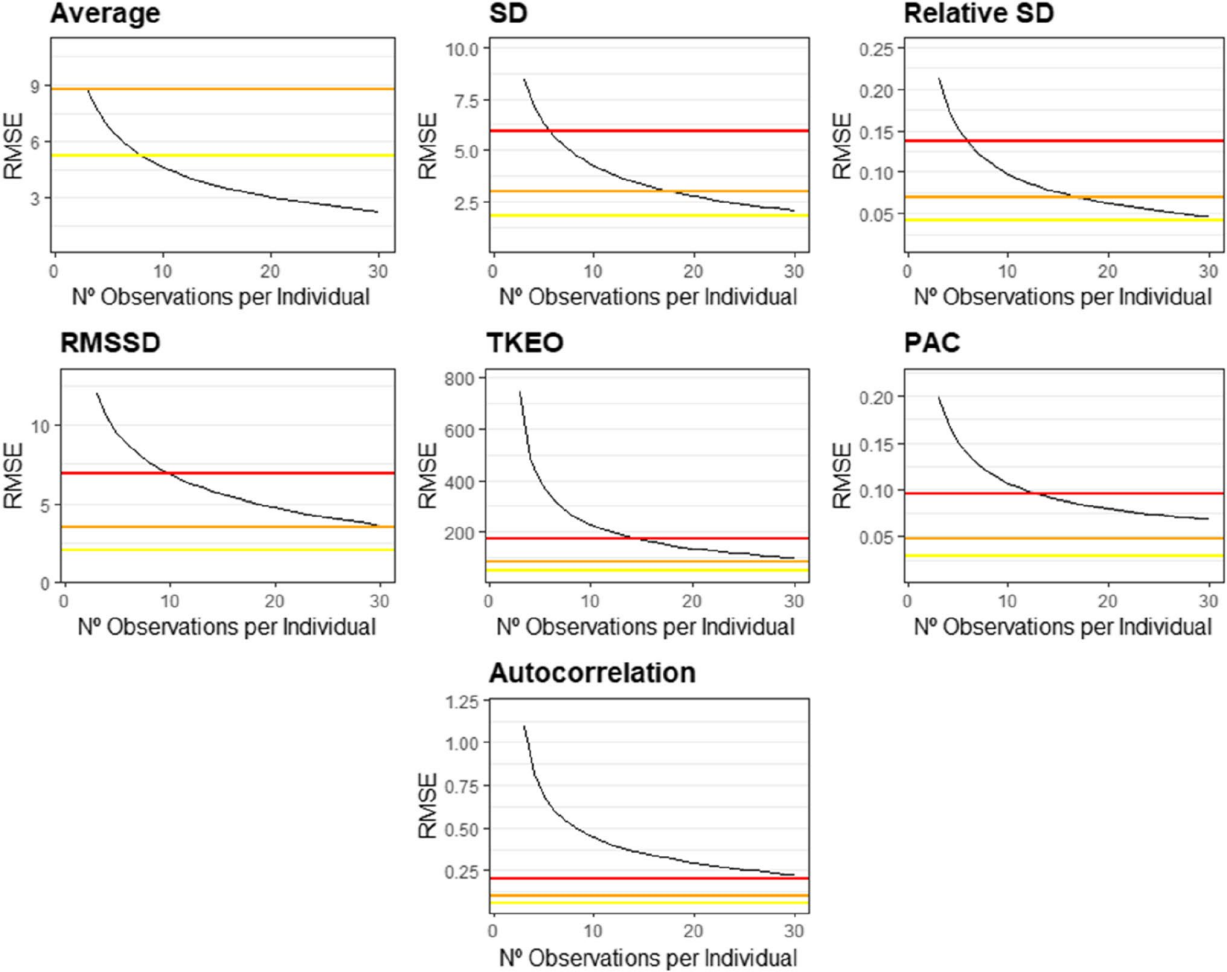
Average RMSE in the estimation of affect dynamics measures as a function of the number of observations per participant. The horizontal lines provide accuracy benchmarks depicting 1 (red), 0.5 (orange), and 0.3 (yellow) between-subjects standard deviations in the affect dynamics measure estimated on the full sample

Figure 3 depicts changes in RMSE as we increase the number of observations per individual used to compute the seven affect dynamics measures. Our results show a large degree of heterogeneity between measures. We found that the number of observations needed to estimate our affect dynamics measures with a minimum accuracy of one between-subject standard deviation in the true measures ranges from 3 for trait affect to over 30 for the autocorrelation coefficient.

### Optimizing sampling approaches

#### Random, close or distant sampling

**Fig. 4:**
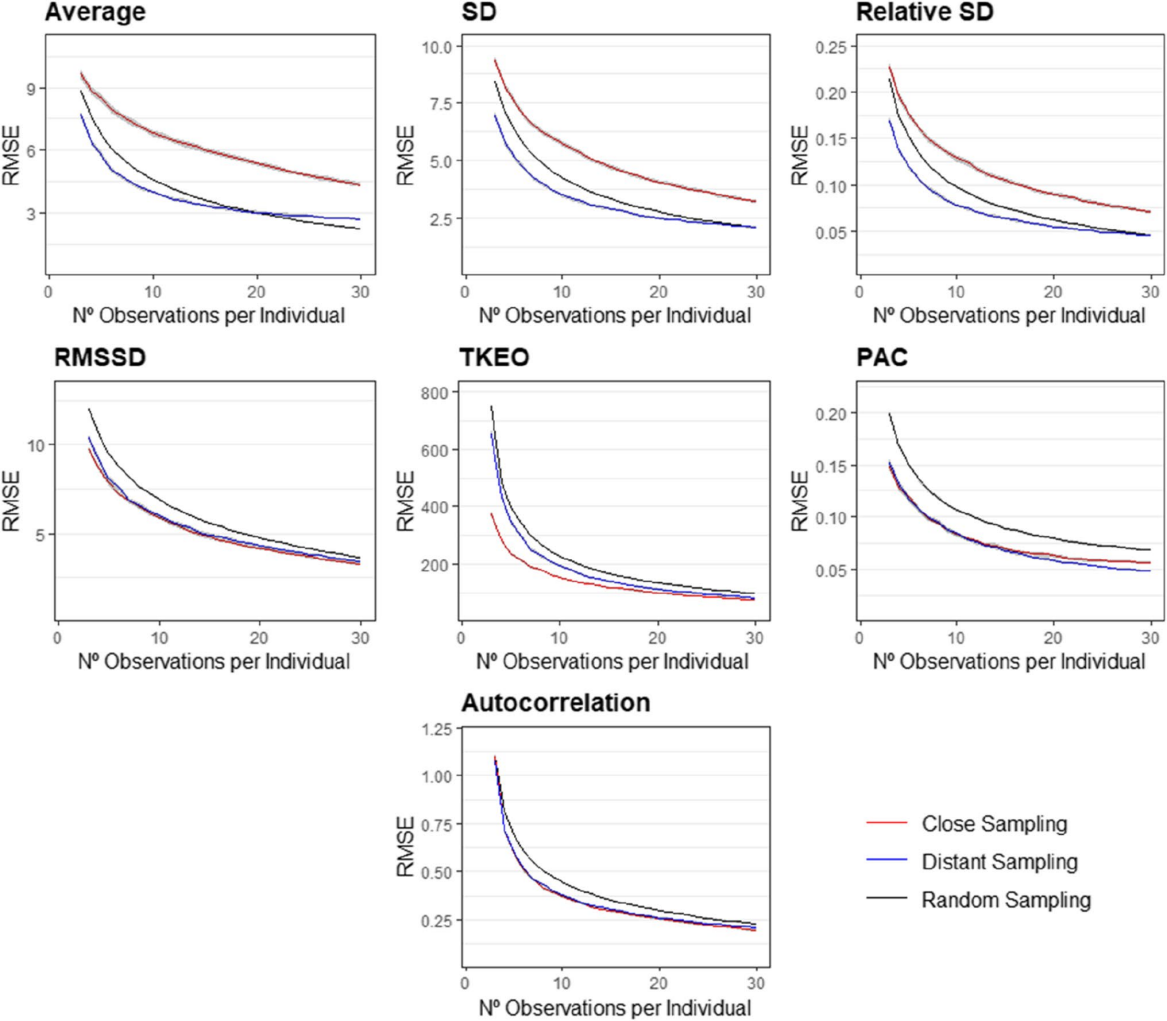
Average RMSE in the estimation of affect dynamics measures as a function of the number of observations per participant collected under random (black), close (red), or distant (blue) sampling. Gray areas around the lines represent the 95% confidence intervals for the average RMSE.

Is it better to conduct short intense studies or longer less-demanding ones? As shown in Fig. 4, the optimal measurement method depends on the affect dynamics measure of interest and the number of observations used to estimate it. We found large differences in the estimation error across sampling methods when calculating affect dynamics measures that are not temporally dependent (i.e., average affect, standard deviation, and relative standard deviation). Estimations of these three measures under close sampling were significantly less accurate than under random and distant sampling. For example, we can estimate a person’s average affect more accurately with ten observations collected at random times over multiple days or weeks than with over 30 consecutive observations over shorter periods of time. In addition, when only a few observations can be collected, we found that distant sampling leads to more accurate estimations than both close and random sampling. Note that the difference between distant and random sampling is small and not statistically significant when at least 27 observations per individual are included in the estimation.

The differences in accuracy across sampling methods were substantially smaller and less consistent for temporally dependent affect dynamics measures (i.e., RMSSD, autocorrelation coefficient, TKEO, and PAC). For RMSSD and the autocorrelation coefficient, estimates obtained through close and distant sampling did not differ, though both of these strategies outperformed random sampling. For TKEO, close sampling largely outperformed both distant and random sampling, especially when the number of observations per participant is small. For the PAC, distant sampling outperformed close and random sampling, especially when the number of observations per participant is large.

#### Random versus specific times sampling

Are there better moments than others to capture people’s affective states? For non-temporally dependent measures (i.e., average affect, standard deviation, and relative standard deviation), random sampling tended to outperform estimates based solely on observations collected at specific times, with estimates based on night hours leading to the highest estimation error (see Supplementary Figures S1–S4). Note that the differences were small and, in many cases, nonsignificant. For affect instability measures (i.e., RMSSD, TKEO, PAC) sampling exclusively at specific times outperforms random sampling, although the differences are small and nonsignificant across most numbers of samples. Sampling earlier in the day, either in the morning or in the afternoon yielded the best results. For affect inertia (i.e., autocorrelation coefficient), sampling exclusively at specific times performed better than random sampling, with estimates based on night hours providing the best performance. Detailed results for random versus specific times sampling can be found in Supplementary Note 2.

#### Random versus specific days sampling

Are there better days than others to capture people’s affect dynamics? For non-temporally dependent measures (i.e., average affect, standard deviation, and relative standard deviation), random sampling tended to perform better than sampling on specific days, with estimates based on weekend observations yielding the highest estimation error. Again, these differences were small and, in many cases, nonsignificant. For measures of affect instability, we did not find differences between random sampling and sampling on specific days for TKEO and PAC, but we found small differences favoring sampling on the weekends for the estimation of the RMSSD. For affect inertia (i.e., autocorrelation coefficient), sampling exclusively on the weekends and sampling exclusively on the weekdays performed better than random sampling, with sampling on the weekends yielding the best performance. Detailed results for random vs. specific days sampling can be found in Supplementary Note 2.

### Statistical power as a function of sampling

**Fig. 5:**
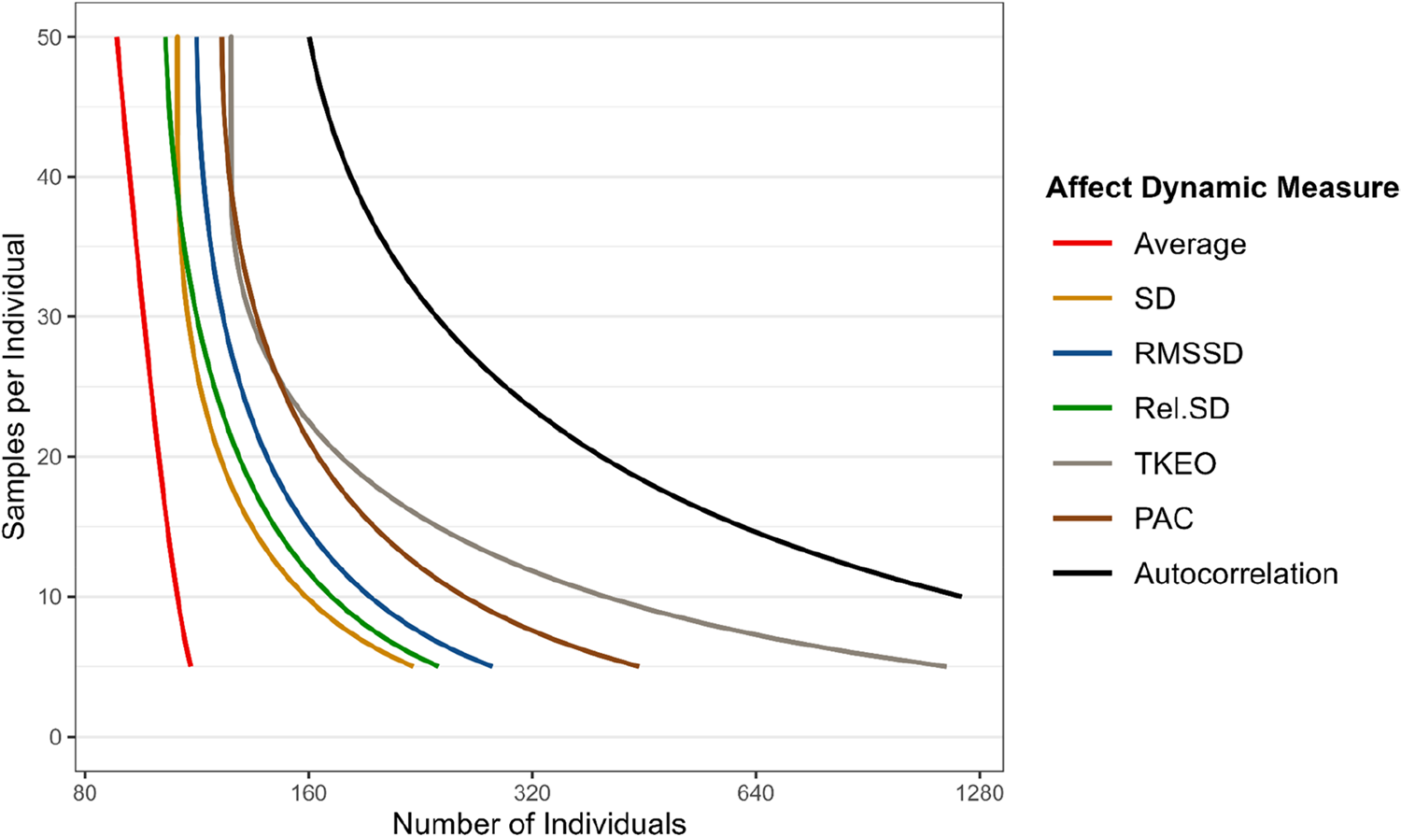
Minimum number of individuals and samples per individual required to achieve sufficient power (≥ 80%) to detect a correlation of medium effect size (*r* = 0.30) with a two-tailed *t*-test and an alpha of 0.05. The *x*-axis is in log-scale

Figure 5 displays the minimal combinations of number of individuals and observations per individual needed to achieve 80% power to detect an association of medium size (*r* = 0.30) using a two-tailed *t*-test and an alpha of 0.05. The different curves are intended to provide a quick overview of how the number of individuals and samples per individuals can be traded off. Detailed information about (1) the method we used to estimate these curves, (2) the specific power achieved for all tested combinations of number of individuals and samples per individual, and (3) other effect sizes and power levels are presented in Supplementary Note 3 and in the online app (https://sergiopirla.shinyapps.io/powerADapp).

Adequate power could be achieved with a relatively small number of observations per individual. As a general rule, as long as a study includes at least 200 participants, sampling 20 observations per individual yields sufficient power for most affect dynamics measures. For average affect, standard deviation, and relative standard deviation, sufficient power was even achieved with 5–10 observations for 200 individuals. For measures of affect instability (i.e., RMSSD, PAC, and TKEO), 20 observations for 200 individuals were required. The only exception to the 200 × 20 rule arises with affect inertia (i.e., autocorrelation coefficient), for which over 40 observations for 200 individuals were required. It is important to note that these sample recommendations apply to studies with an expected medium-sized association of interest (*r* = 0.30). However, as our plausible effect sizes benchmarks suggest, many affect dynamics measures display relatively weak associations with demographic, well-being, and time-allocation outcomes (see next section).

Overall, averaging across the range of all sampling combinations, affect dynamics measures, alpha levels, and effect sizes, increasing the number of individuals had a larger impact on power than increasing the number of observations per individual—with the exception of affect inertia which showed the opposite pattern (see Supplementary Note 4).

### Benchmarks for plausible effect sizes

**Fig. 6:**
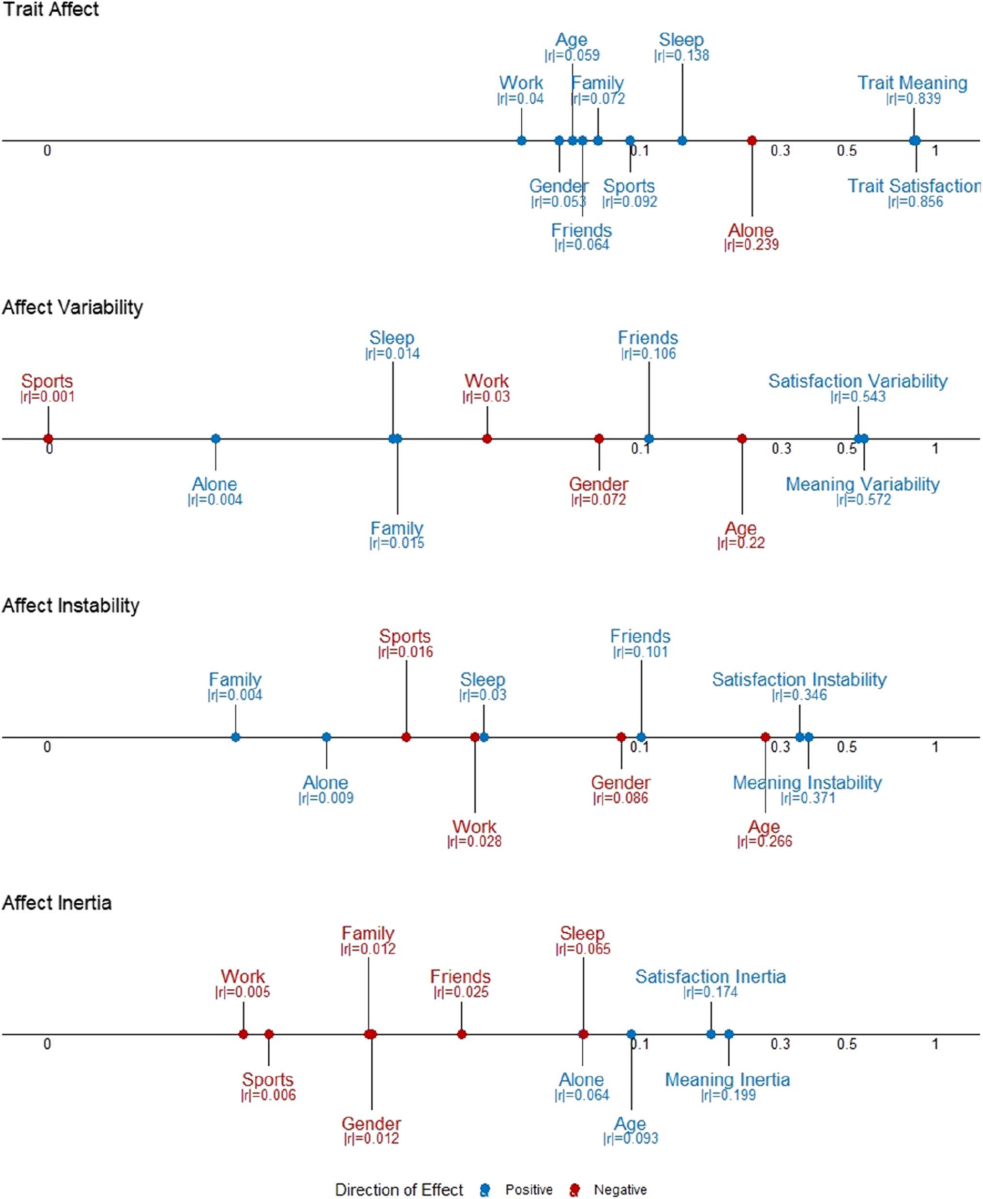
Correlations between affect dynamics measures and different outcome variables in our dataset. Positive and negative correlations are presented in blue and red, respectively

In power calculation, researchers are asked to anticipate the effect sizes of their associations of interest or to decide on a minimum effect size that they are willing to detect. How can one know in advance what plausible effect sizes might be? Fig. 6 displays the magnitude of the associations between affect dynamics measures and ten outcomes: (1) age, (2) gender, (3) average sleep time, (4) life satisfaction, (5) meaning in life, as well as the proportion of time spent with (6) friends, (7) family, (8) alone, (9) working, and (10) exercising. These values can be used as broad benchmarks when attempting to postulate plausible effect sizes (see Supplementary Note 5 for additional information and results). For example, researchers interested in examining the relationship between average affect and the propensity to eat carrots could ask themselves whether they expect this relationship to be smaller or greater than the link between average affect and age (*r* = 0.06), time spent alone (*r* =  − 0.24), or trait meaning in life (*r* = 0.84). Likewise, researchers interested in examining the relationship between affect instability and family history of bipolar disorder could ask themselves whether they expect the relationship to be smaller or greater than the link between affect instability and time spent with friends (*r* = 0.10), age (*r* =  − 0.27), or life satisfaction instability (*r* = 0.34). In practice, researchers should not exclusively rely on these benchmark effect sizes to establish an expected effect size but consider information from different sources (including meta-analytic evidence, preliminary results, or past literature). These benchmarks thus provide a useful complementary source of information to help in defining an expected effect size.

### R package and online power calculator:

Building on our results and expanding our power calculations to all effect sizes, we developed an R package (“powerAD”) and a Shiny app (https://sergiopirla.shinyapps.io/powerADapp/) to help researchers make empirically informed decisions about study design of affect dynamics studies. We refer to the package site (https://sergiopirla.github.io/powerAD) for more information on how to download, install, and run its primary functions.

**Fig. 7:**
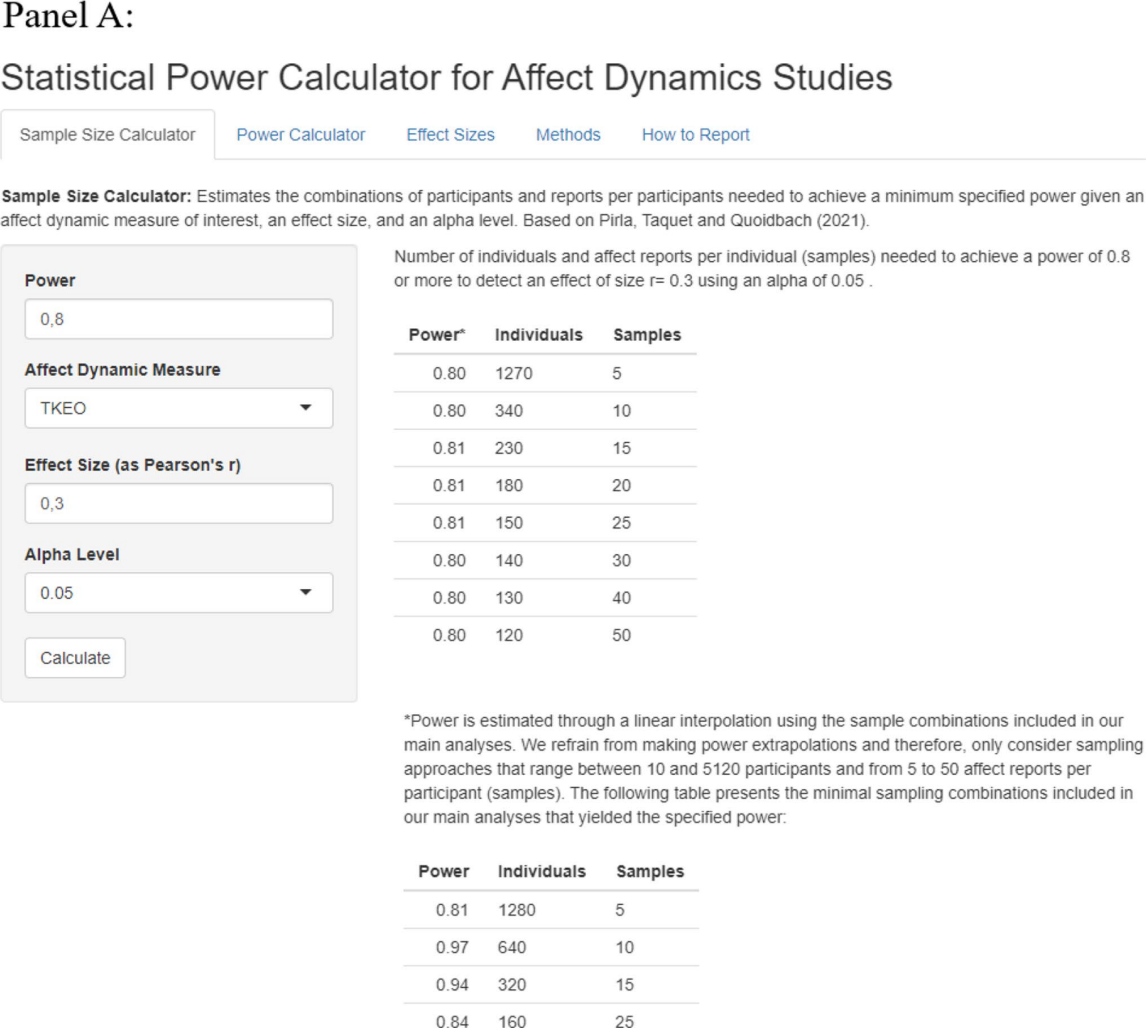

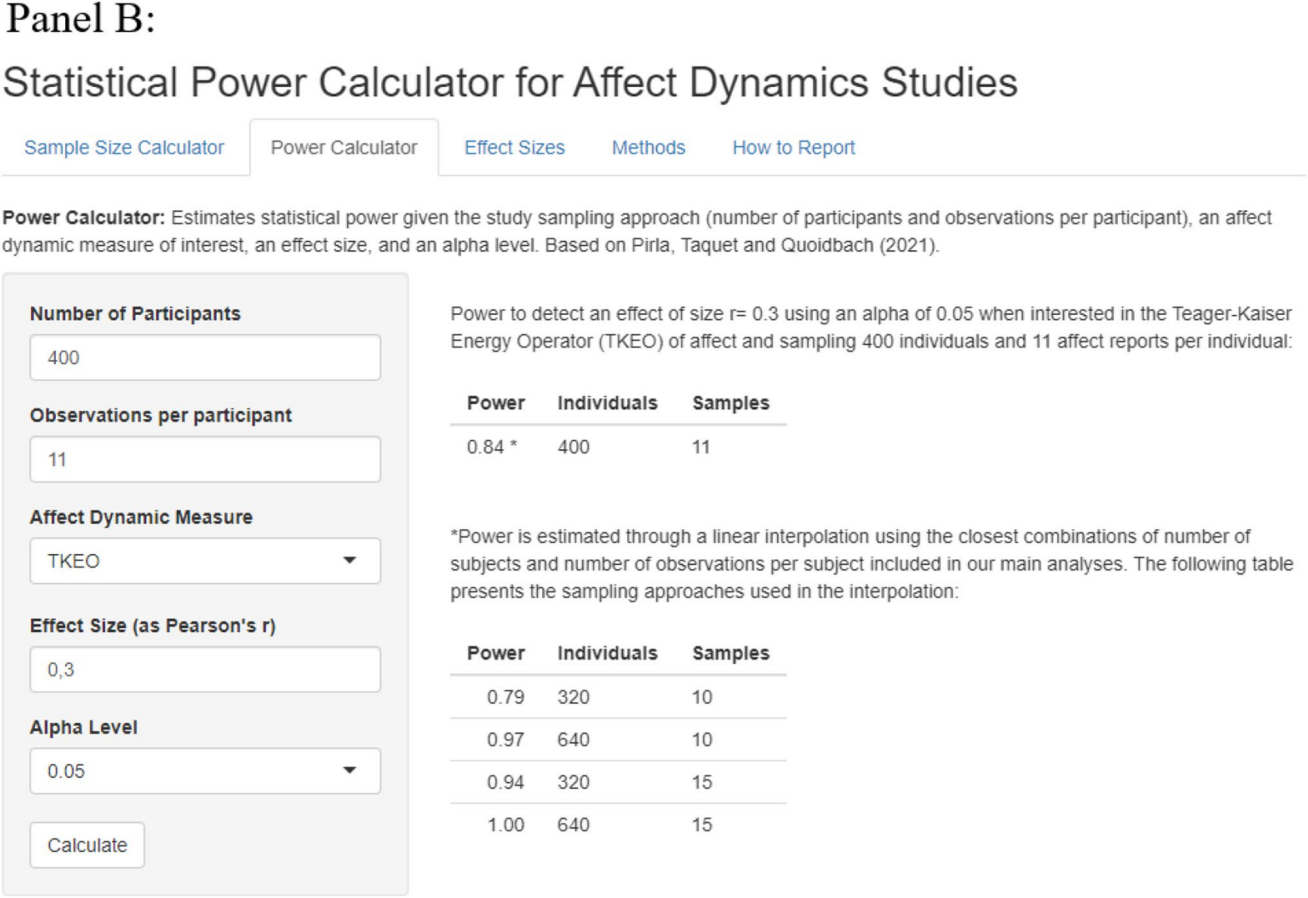
Shiny app to calculate power in affect dynamics studies. Panel A shows the sample size calculator. Panel B shows the power calculator

Our Shiny app is composed of two main panels. On the first panel (“sampling calculator”), users can estimate a set of valid sampling approaches for each affect dynamics measure given a specified statistical power, effect size, and alpha level. On the second panel (“power calculator”), users can estimate the statistical power achieved by a specific study based on its characteristics (sampling approach, affect dynamics measure, effect size, and alpha level). For example, panel A of Fig. 7 shows the minimal combinations of number of individuals and number of observations per individual to obtain a statistical power of 80% to detect an *r* = 0.30 at the 5% significance level for the Teager–Kaiser energy operator (TKEO). Panel B provides the precise power estimate for the same *r* = 0.30 effect size and TKEO measure given a specific sample of 400 participants, each surveyed 11 times. Finally, the app also provides a series of benchmark effect sizes for each affect dynamics measure to help researchers estimate plausible effect sizes.

## Discussion

This paper introduces an empirically derived framework to help researchers design well-powered and efficient experience sampling studies in the growing field of affect dynamics. To illustrate the value of this contribution, imagine that a group of researchers want to design an ESM study examining the association between affect variability and burnout risk. Using the online tool (“Effect sizes” tab) they anticipate that the effect size should be in the same ballpark as the relationship between affect variability and average life satisfaction (which, using our benchmarks, they observe to be *r* = 0.20). Using the “Sample Size Calculator” tab and setting the power to 0.80, the effect size to 0.20, and the alpha level to 0.05, they notice that they have a range of options to achieve this power. For instance, they could recruit 240 participants and collect 40 affect records from each or they could recruit 510 participants and collect five affect records from each. Because they are mindful that retention of participants can be an issue, they opt for the latter option.

Whereas the ideal sampling approach depends on the specific affect dynamics measure under consideration, three design principles emerge from our research. First, a sample of 200 participants each providing 20 observations (i.e., 200 × 20 rule) yields sufficient power to detect medium-sized associations for most affect dynamics measures. Second, the optimal sampling strategy depends on the affect dynamics measure of interest. For trait affect and affect variability, it is often better to run longer less-demanding studies (i.e., few daily measurements spread out over several weeks) than shorter intense ones (i.e., many daily affect measurements spread out over several days). For measures of instability and inertia, both short intense studies and longer less-demanding studies outperform random samples with little difference between the two designs. Third, little differences were observed between random sampling and sampling at specific times or on specific days, so that the choice of sampling moments can be dictated by other considerations (such as the individual’s preferences or practicalities related to the study at hand).

The present study provides a robust empirical framework to conduct ESM studies in affective science. But it is important for future research to address several limitations. First, our “true” values (i.e., those based on all the available measurements for an individual) were based on at least 50 observations per participant. It might be that more extensive data at the participant level (e.g., 1000 observations per individual) would lead to somewhat different inferences. Second, our recommendation about when researchers should survey participants is limited to relatively basic strategies (e.g., random moments vs. specific days or times). Future research is needed to examine whether advanced context-aware strategies (e.g., sending surveys in response to changes in participants' environmental or psychological circumstances) lead to substantial gains in accuracy and statistical power. Third, although we relied on an exceptionally large sample, our participants may not be representative of the general population. Future research is also needed to examine whether our recommendations need to be adjusted for specific groups of people (e.g., patients with depression, older adults). Fourth, our recommendations are based on accuracy and statistical power considerations. They do not take into account how different sampling strategies may affect burden, compliance, and careless responding in ESM research. Our data did not include information on non-answered notifications, limiting our ability to test the impact of our sampling recommendations on burden and compliance. While recent research suggests that sampling frequency has no impact on participant’s burden, data quantity, and data quality (Eisele et al., 2020), further research is needed to examine whether other recommendations derived from our findings are similarly free of negative consequences. Finally, our framework focused on a general, unidimensional measure of affect (unhappy–happy) and the optimal sampling strategies to detect correlations. In future research, it is important to examine how different affect measurements impact estimation precision and statistical power. Further work should also explore how our recommendations apply to other affective states, including specific emotions, mixed-effects models, and nonlinear relationships between affect dynamics measures and outcomes. We hope that the data and code provided will allow researchers to expand our framework, opening the door to fast and exciting advances in the study of human emotions.

## Supplementary Information

Below is the link to the electronic supplementary material.

Supplementary file1 (DOCX 481 kb)
