# Supplementary material for: Measuring affect dynamics: An empirical framework
Source: Behav Res Methods. 2022 Apr 5;55(1):285–300. doi: 10.3758/s13428-022-01829-0 (PMC9918585; doi:10.3758/s13428-022-01829-0)
Supplement: Supplementary file 1 — Supplementary file1 (DOCX 481 kb) [file 13428_2022_1829_MOESM1_ESM.docx]

**Supplementary Materials**

**Note 1: Sample summary statistics**

The following tables present the summary statistics for the sample of participants included in our analyses:

| Sample variable | Average | SD | Min | Max |
| --- | --- | --- | --- | --- |
| Age | 29.97 | 9.96 | 13 | 73 |
| Number of affect reports per participant | 111.55 | 87.79 | 51 | 1689 |

**Table S1** Sample summary statistics, numeric variables. *n* = 7016.

| Sample variable | Proportion |
| --- | --- |
| Gender (female) | 73.82% |
| Gender (male) | 26.18% |
| Country (France) | 92.63% |
| Country (Switzerland) | 4.83% |
| Country (Belgium) | 0.59% |
| Country (Other) | 0.37% |

**Table S2** Sample summary statistics, categorical variables. *n* = 7016.

| Sample variable | Proportion |
| --- | --- |
| Morning (from 6 a.m. to 12 p.m.) | 31.15% |
| Afternoon (from 12 p.m. to 4 p.m.) | 25.16% |
| Evening (from 4 p.m. to 8 p.m.) | 27.72% |
| Night (from 8 p.m. to 6 a.m.) | 15.85% |

**Table S3** Temporal distribution of affect reports.

**Note 2: Optimizing sampling approaches**

In this section we present in greater detail the results obtained when optimizing sampling based on specific times and days. To compare performance across time windows, we followed these steps: For a given individual and number of reports between 3 and 30, (1) we resampled without replacement and estimated affect dynamics measure 1000 times, (2) we applied a debiased step to each of these 1000 estimates, (3) we estimated an individual’s root mean square error (RMSE) for each number of reports based on the full sample “true” estimates of affect dynamics measures, and (4) we averaged the RMSE across participants for each number of reports used in the computation of the affect dynamics measures. To debias the estimates, we first obtained a condition-specific bias by subtracting from the average of the population of true values of an affect dynamics measure the average of the population of the same measure estimated with affect reports from our time window of interest. We then subtracted this bias to each of our estimates of affect dynamics measures obtained from resampling 1000 times. For example, when analyzing the performance of the estimations of the TKEO with reports collected at night, we first obtained a condition-specific bias. To calculate this bias term, we (1) estimated each individual’s TKEO in affect using all reports available, (2) estimated each individual’s TKEO in affect using all reports collected at night, (3) averaged (1) and (2) across individuals, and (4) subtracted from the average of the population of TKEOs estimated with all reports the average of the population of TKEOs estimated with the reports collected at night. This debiasing procedure allowed us to account for “condition fixed effects,” any constant bias across individuals that did not affect the relative ordering of individuals in terms of their affect dynamics measure of interest. For completeness, we also present the results of the analyses without a debiasing step. Including the debiasing step did not substantially change our results. The results are presented in the Supplementary Figures S1–S4. Figure S5 presents the results obtained when considering a sampling strategy that includes at least one observation from each time window. The estimations of performance in this last condition did not include a debiasing step. We followed the same procedure outlined here to compare performance when sampling across specific days. Supplementary Figures S6 and S7 present the results obtained when sampling on specific days.

**
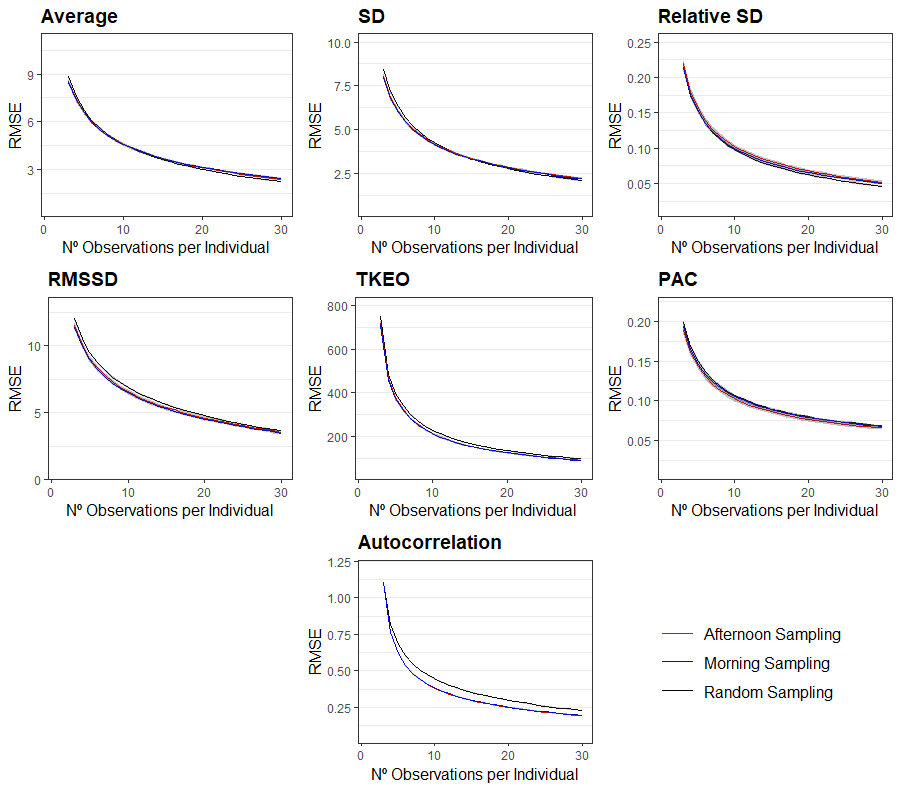
**

**Figure S1** Between-subject mean RMSE for affect dynamics measures as a function of time of the day. Random times are depicted in black, morning (6 a.m. to noon) in blue, and afternoon (noon to 4 p.m.) in red. Gray areas around the lines represent the 95% confidence intervals for the average RMSE. These estimates are calculated after a debiasing step.


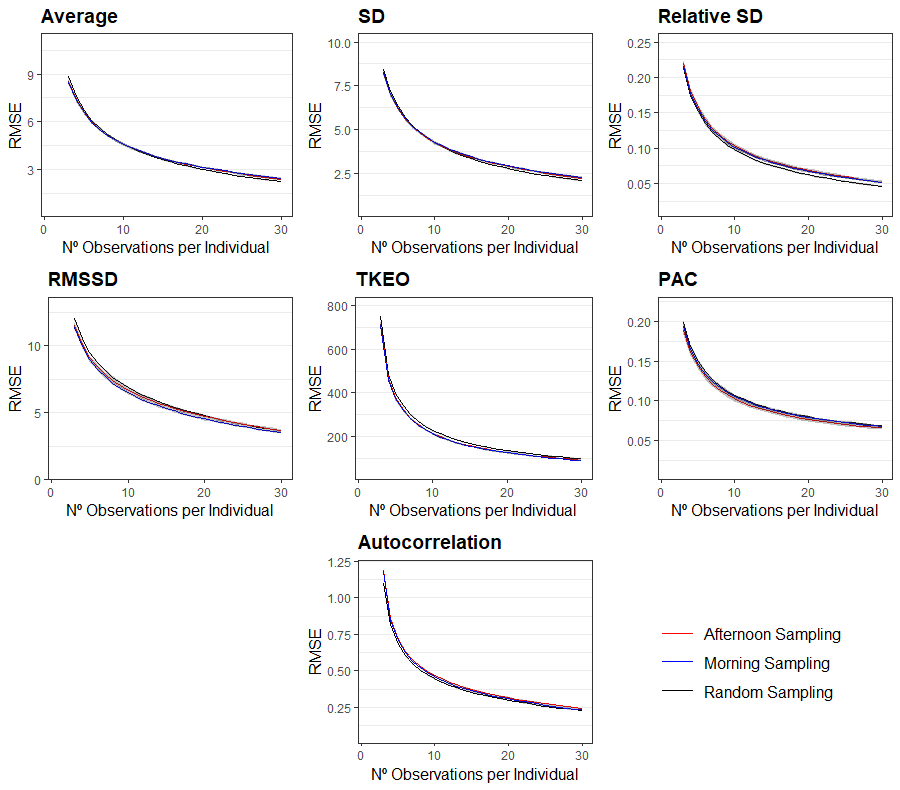


**Figure S2** Between-subject mean RMSE for affect dynamics measures as a function of time of the day. Random times are depicted in black, morning (6 a.m. to noon) in blue, and afternoon (noon to 4 p.m.) in red. Gray areas around the lines represent the 95% confidence intervals for the average RMSE. These estimates are calculated without a debiasing step.


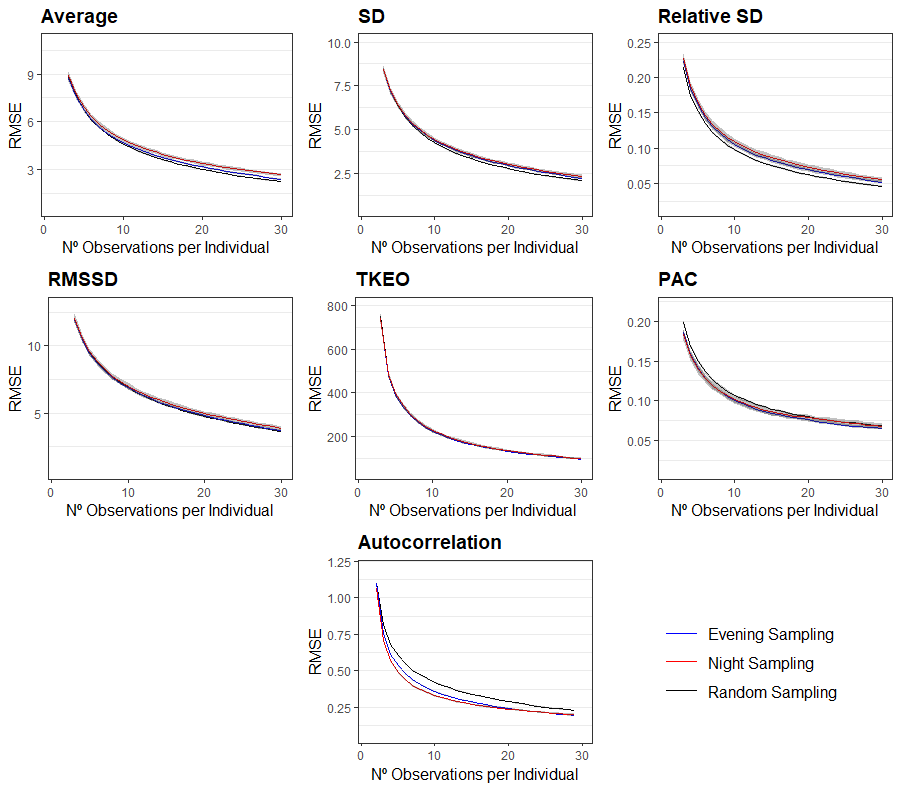


**Figure S3** Between-subject mean RMSE for affect dynamics measures as a function of time of the day. Random times are depicted in black, evening (4 p.m. to 8 p.m.) in blue, and night (8 p.m. to 6 a.m.) in red. Gray areas around the lines represent the 95% confidence intervals for the average RMSE. These estimates are calculated after a debiasing step.


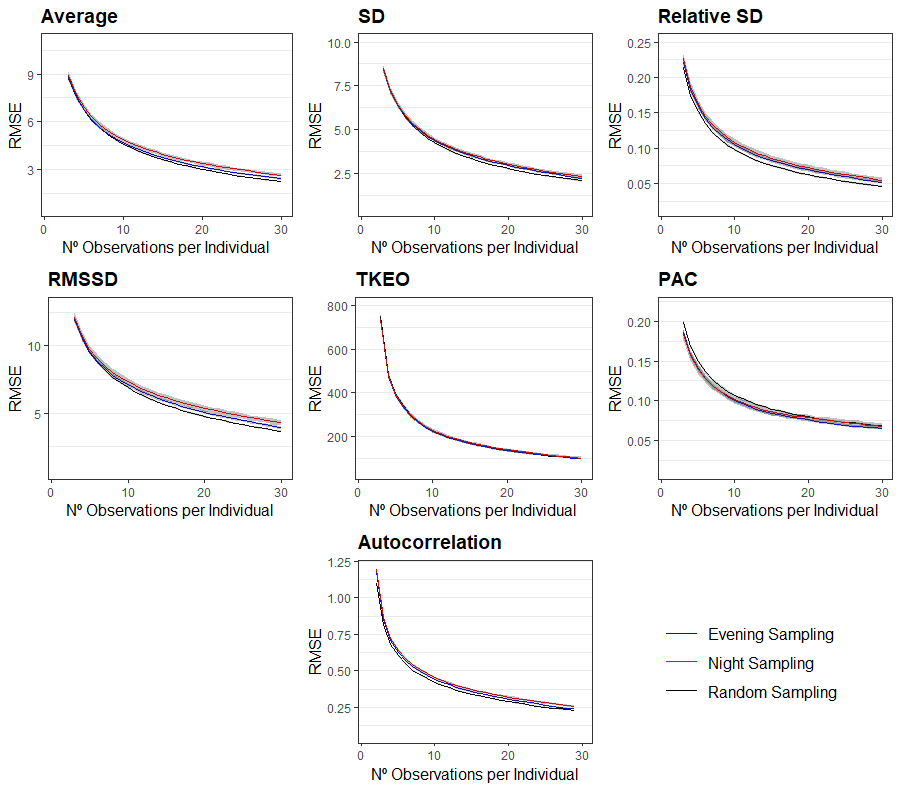


**Figure S4** Between-subject mean RMSE for affect dynamics measures as a function of time of the day. Random times are depicted in black, evening (4 p.m. to 8 p.m.) in blue, and night (8 p.m. to 6 a.m.) in red. Gray areas around the lines represent the 95% confidence intervals for the average RMSE. These estimates are calculated without a debiasing step.


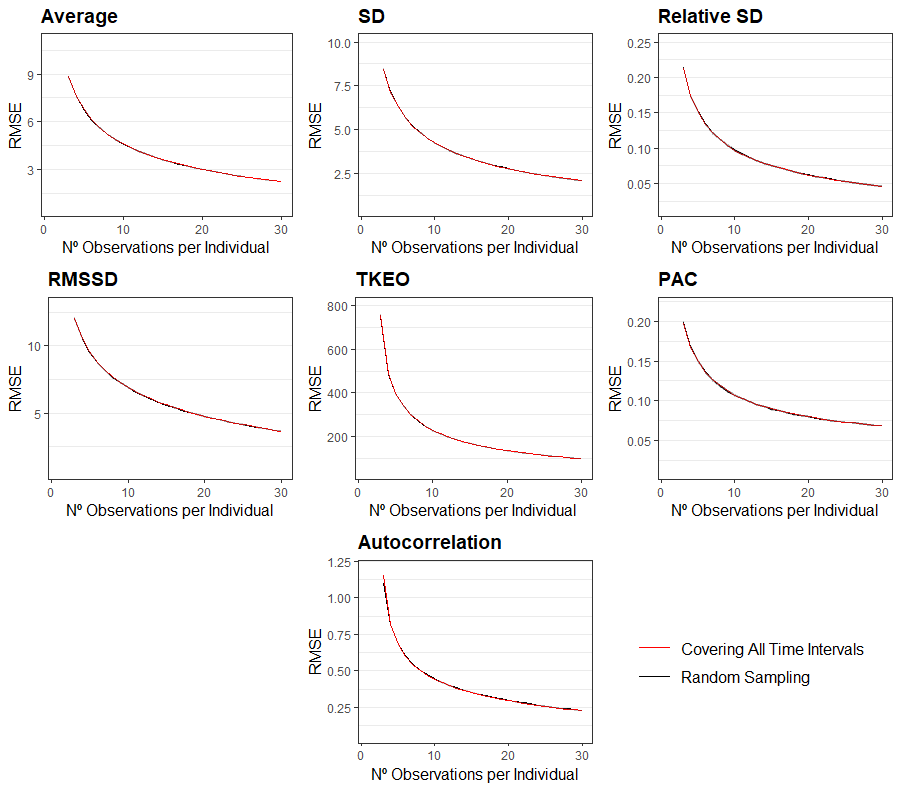


**Figure S5** Between-subject mean RMSE for affect dynamics measures as a function of time of the day. Random times are depicted in black, and estimates obtained when sampling a minimum of one observation from each time interval (morning, afternoon, evening and night) in red. Gray areas around the lines represent the 95% confidence intervals for the average RMSE.


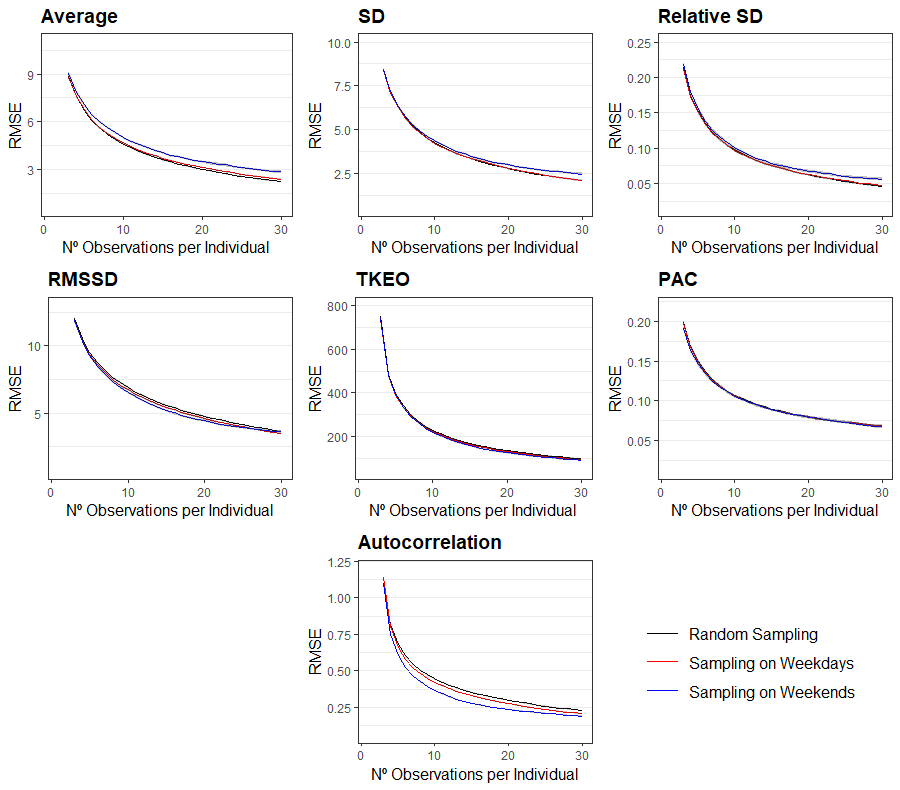


**Figure S6** Between-subject mean RMSE for affect dynamics measures as a function of day of the week. Random days are depicted in black, weekends in blue, and weekdays (8 p.m. to 6 a.m.) in red. Gray areas around the lines represent the 95% confidence intervals for the average RMSE. These estimates are calculated after a debiasing step.


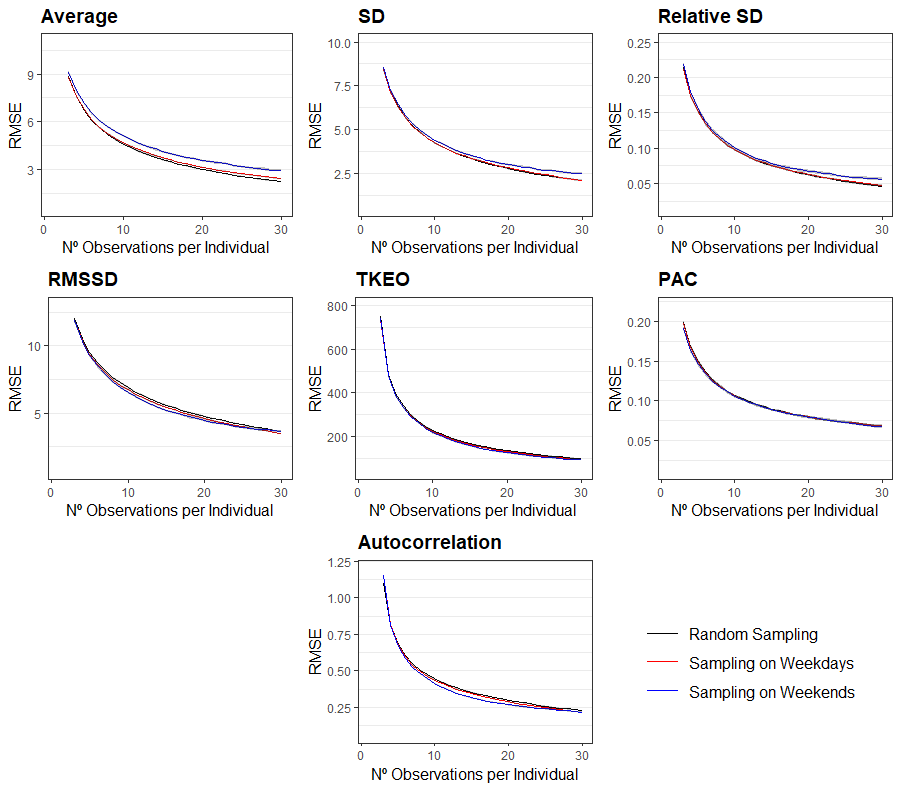


**Figure S7** Between-subject mean RMSE for affect dynamics measures as a function of day of the week. Random times are depicted in black, weekends in blue, and weekdays (8 p.m. to 6 a.m.) in red. Gray areas around the lines represent the 95% confidence intervals for the average RMSE. These estimates are calculated without a debiasing step.

**Note 3: Statistical power as a function of sampling**

In this note, we first detail the procedure followed to obtain Fig. 5 of the main text and then present empirical power for all sampling combinations included in our study when interested in weak (*r* = .10), medium-sized (*r* = .30), or strong correlations (*r* = .50).

Figure 5 presents the minimum number of individuals and number of samples per individual for each affect dynamics measure to yield 80% power to detect a medium-sized relationship (*r* = .30) with an alpha of 0.05. To estimate the curves, we used the results of the empirical power estimations obtained in our main analyses (presented below in this Note). Using these results, for each affect dynamics measure, we first focused on those analyses that assumed an alpha of 0.05 and a medium-sized correlation (*r* = .30). Then, for each number of samples per participant in our analyses (that is, from 5 to 50 in increments of 5), we selected the minimum number of participants that yielded a power larger than or equal to 80%, and the maximum number of participants that yielded a power lower than or equal to 80%. We divided the space between these two numbers of participants into a sequence with increments of 10 participants and linearly interpolated power for each number of participants included in this sequence. Using these interpolated power values, we selected, for each number of samples per participant, the minimum number of individuals with an approximated power larger than or equal to 80%. This process yielded, for each affect dynamics measure, 10 combinations of individuals and number of samples per participants with an approximate power over 80%. As small variations existed in power (approximated power ranged between 80 and 83%) and we relied on linear approximations, we encountered some non-monotonic regions—that is, regions where the number of participants needed to achieve a minimum power of 80% did not decrease with the number of samples per participant but displayed minor increases. We directly imposed weak monotonicity by replacing these regions by sequences in which the number of participants remained unchanged as the number of samples per participant was increased. In doing so, for each affect dynamics measure we obtained our 10 final sampling combinations with an approximate power of 80%. Using these 10 combinations, we regressed, for each affect dynamics measure, the logarithm of the number of participants on the number of samples per participant and its logarithm. This specification was selected based on fit—the average R-squared across affect dynamics measures was above 95%. We used the fitted number of participants for each number of samples per participant to draw the curves. Supplemental Table S4 presents the final 10 sampling combinations used to run the regression models for each affect dynamics measure. We abstain from making power extrapolations, and therefore, for affect inertia (“Auto.”), we do not provide the number of individuals needed when sampling affect five times from each participant. This number would be well above 5120 participants.

| Samples | Number of individuals | | | | | | |
| --- | --- | --- | --- | --- | --- | --- | --- |
|  | Average | SD | Rel. SD | RMSSD | TKEO | PAC | Auto. |
| 5 | 120 | 230 | 260 | 300 | 1200 | 460 | - |
| 10 | 110 | 150 | 160 | 200 | 360 | 260 | 1690 |
| 15 | 110 | 130 | 140 | 160 | 240 | 210 | 810 |
| 20 | 100 | 130 | 130 | 140 | 180 | 160 | 510 |
| 25 | 100 | 120 | 120 | 140 | 170 | 160 | 400 |
| 30 | 90 | 110 | 110 | 130 | 150 | 140 | 290 |
| 35 | 90 | 110 | 110 | 120 | 140 | 140 | 250 |
| 40 | 90 | 110 | 110 | 120 | 140 | 130 | 210 |
| 45 | 90 | 110 | 110 | 120 | 130 | 130 | 190 |
| 50 | 90 | 100 | 110 | 120 | 130 | 130 | 160 |

**Table S4** Sampling combinations yielding an approximate power of 80% to detect a medium-sized association (*r* = .30) with an alpha of 0.05 for each affect dynamics measure. “Samples” represent the number of samples per participant needed and “number of individuals” the total number of participants.

Next, we focused on the results of estimating the empirical power for weak (*r* = .10) and strong correlations (*r* = .50). To estimate power, we followed the same procedure presented in the main text. We took a conservative approach and set the type I error rate at 0.001.


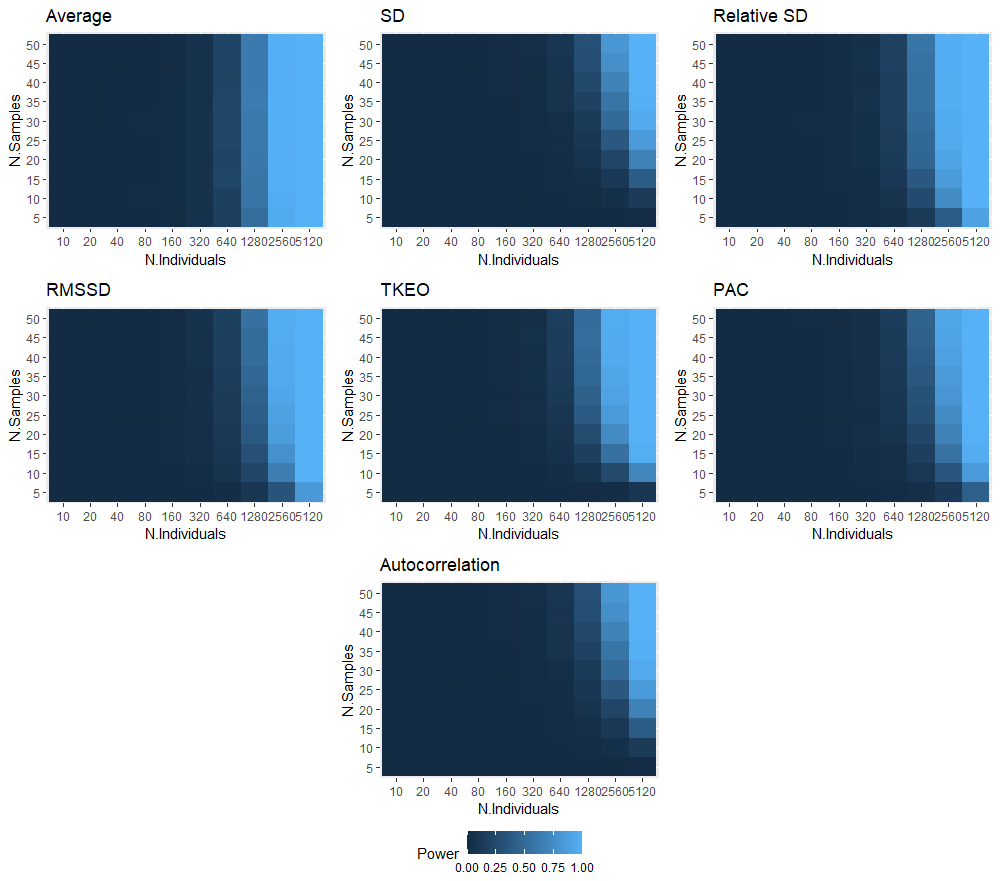


**Figure S8** Power as a function of sampling strategy. Each panel represents the power to detect a small correlation (*r* = .10) between an affect dynamics measure and an outcome variable using a two-tailed *t*-test and an alpha of 0.001.

*
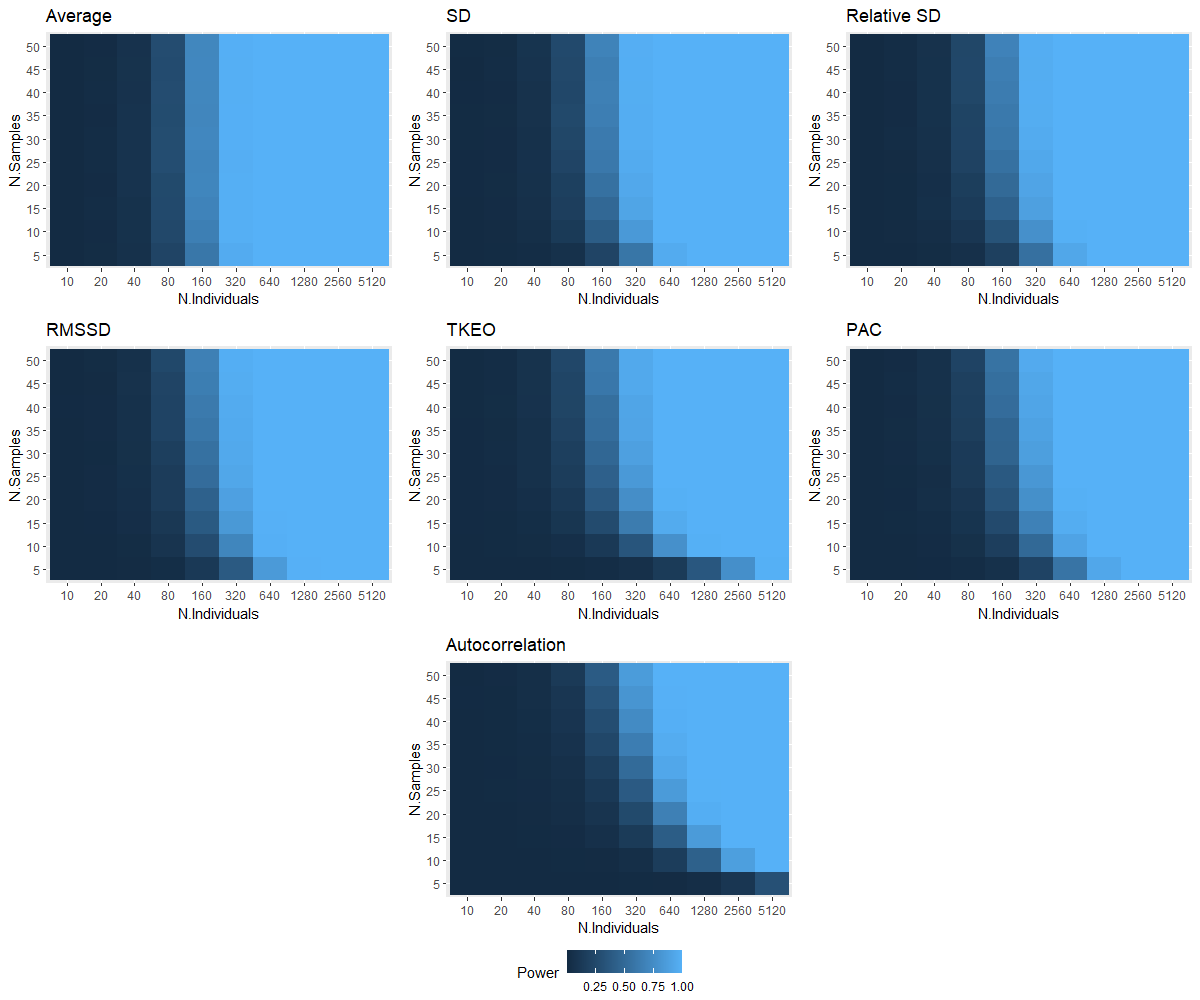
*

**Figure S9** Power as a function of sampling strategy. Each panel represents the power to detect a small correlation (*r =*.30) between an affect dynamics measure and an outcome variable using a two-tailed *t*-test and an alpha of 0.001.


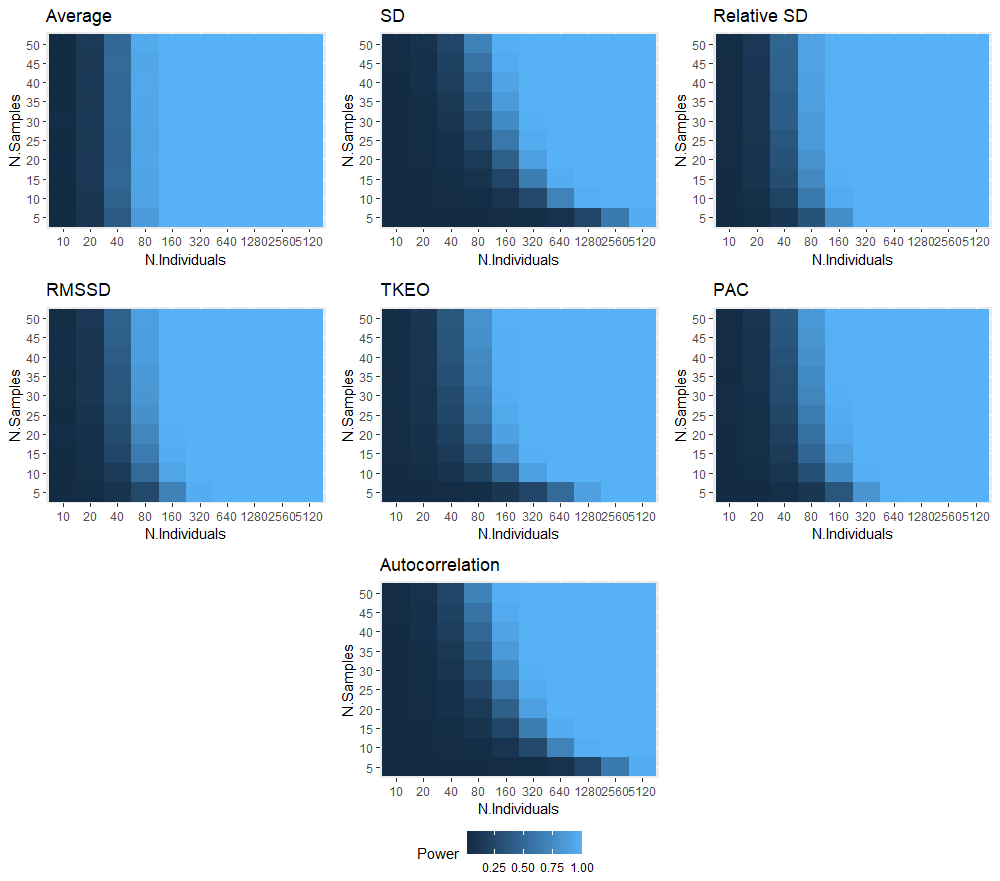


**Figure S10** Power as a function of sampling strategy. Each panel represents the power to detect a small correlation (*r =*.50) between an affect dynamics measure and an outcome variable using a two-tailed *t*-test and an alpha of 0.001.

**Note 4: Average effect of number of participants and samples on power**

In the following tables we present the results of regressing empirical power on the number of individuals and samples per participant for all affect dynamics measures. Note that our sample size for each measure is 1500 observations, as we include power obtained from our 100 sampling combinations, three effect sizes (small, medium and large), and five alpha levels (0.1, 0.05, 0.01, 0.005, 0.001).

|  | Power  (all measures) | Power  (average) | Power  (SD) | Power  (Rel. SD) |
| --- | --- | --- | --- | --- |
| log(Individuals) | 0.156*** | 0.153*** | 0.157*** | 0.157*** |
|  | (0.001) | (0.003) | (0.003) | (0.003) |
| log(Samples) | 0.080*** | 0.008 | 0.045*** | 0.048*** |
|  | (0.004) | (0.010) | (0.010) | (0.010) |
| Constant | −0.525*** | −0.211*** | −0.379*** | −0.394*** |
|  | (0.014) | (0.037) | (0.037) | (0.037) |
| Observations | 10,500 | 1500 | 1500 | 1500 |
| *R*^2^ | 0.567 | 0.562 | 0.583 | 0.585 |

**Table S5** Ordinary least squares (OLS) estimates of the effect of number of individuals and samples per participant on statistical power across effect sizes and significance levels. Standard errors are in parentheses. Statistical significance: **p* < 0.1; ***p* < 0.05; ****p* < 0.01

|  | Power  (RMSSD) | Power  (TKEO) | Power  (PAC) | Power  (Auto.) |
| --- | --- | --- | --- | --- |
| log(Individuals) | 0.160*** | 0.158*** | 0.160*** | 0.147*** |
|  | (0.003) | (0.003) | (0.003) | (0.003) |
| log(Samples) | 0.058*** | 0.128*** | 0.079*** | 0.196*** |
|  | (0.010) | (0.010) | (0.010) | (0.010) |
| Constant | −0.454*** | −0.709*** | −0.551*** | −0.974*** |
|  | (0.036) | (0.036) | (0.037) | (0.036) |
| Observations | 1500 | 1500 | 1500 | 1500 |
| *R*^2^ | 0.596 | 0.612 | 0.596 | 0.598 |

**Table S6** Ordinary least squares (OLS) estimates of the effect of number of individuals and samples per participant on statistical power across effect sizes and significance levels. Standard errors are in parentheses. Statistical significance: **p* < 0.1; ***p* < 0.05; ****p* < 0.01

**Note 5: Plausible effect sizes**

We collected our data using “58 seconds,” a free smartphone application. At sign-up, the participants answered a few questions regarding demographic information. Using such information, we coded the variable “gender” to take a value of 1 for male participants and 0 for female participants. The numeric variable “age” represents a participant’s age in years. Data on life meaning, satisfaction, sleep, and proportion of time spent with different groups of people or engaging in different activities was collected using a system of random notifications. Participants using the app received questionnaire prompts at random times of the day. These questionnaires consisted of four to six questions from a large battery of items. For meaning in life, the participants were asked to rate the following statement from 0 (“Not at all”) to 100 (“Absolutely”): “Here and now I feel like I'm living a meaningful life.” Similarly, for life satisfaction, participants provide a rating from 0 (Dissatisfied with my life) to 100 (Satisfied with my life) to the following statement: “Here and now, I feel…” For life meaning as for life satisfaction, we used these numeric reports to estimate the seven dynamic measures included in this paper (average, SD, Rel. SD, RMSSD, TKEO, PAC and autocorrelation). Sleep was measured by asking participants the amount of sleep hours they had last night. Participants provided a numeric report ranging from 0 to 15, and we averaged across sleep reports to obtain an individual’s average hours of sleep. Finally, some questionnaires included a list of activities and a list of groups of people. The participants facing these lists were asked to select all activities that they were doing before answering the questionnaire and select all the groups of people with whom they were when answering the questionnaire. For simplicity, we restricted our attention to the proportion of time an individual spent with family, friends, alone, studying or working, and exercising. To obtain proxies for the amount of time that an individual spent with these people or doing these activities, we estimated the proportion of times (out of all times that the participant was presented with each list) that the user reported being with the specific group of people or doing a specific activity.

For each participant, aside from these variables, we estimated the seven affect dynamics measures included in our main analyses using the full sample of affect reports at our disposal. To provide the reader with effect sizes to serve as reference, we estimated the Pearson’s *r* coefficient between each affect dynamics measure and the demographic, well-being, and time allocation variables described in the previous paragraph. The resulting coefficients are presented in Supplemental Table 7.

| Outcome | Average | SD | Rel. SD | RMSSD | TKEO | PAC | Auto. |
| --- | --- | --- | --- | --- | --- | --- | --- |
| Age | 0.059 | −0.248 | −0.191 | −0.282 | −0.247 | −0.269 | 0.093 |
| Gender | 0.053 | −0.098 | −0.047 | −0.093 | −0.081 | −0.085 | −0.012 |
| Average Meaning in life | 0.839 | −0.231 | 0.096 | −0.201 | −0.194 | −0.236 | −0.029 |
| SD Meaning in life | 0.019 | 0.520 | 0.431 | 0.468 | 0.452 | 0.413 | 0.055 |
| Rel. SD Meaning in life | 0.144 | 0.414 | 0.623 | 0.365 | 0.373 | 0.318 | 0.059 |
| RMSSD Meaning in life | 0.014 | 0.472 | 0.387 | 0.469 | 0.448 | 0.422 | −0.031 |
| TKEO Meaning in life | 0.039 | 0.295 | 0.280 | 0.282 | 0.284 | 0.249 | 0.005 |
| PAC Meaning in life | 0.040 | 0.381 | 0.355 | 0.387 | 0.383 | 0.360 | −0.041 |
| Auto. Meaning in life | 0.017 | 0.016 | 0.026 | −0.076 | −0.058 | −0.082 | 0.199 |
| Average Life satisfaction | 0.856 | −0.197 | 0.109 | −0.144 | −0.144 | −0.181 | −0.080 |
| SD Life satisfaction | −0.076 | 0.508 | 0.393 | 0.439 | 0.428 | 0.408 | 0.096 |
| Rel. SD Life satisfaction | 0.141 | 0.363 | 0.579 | 0.323 | 0.329 | 0.286 | 0.053 |
| RMSSD Life satisfaction | −0.087 | 0.471 | 0.353 | 0.447 | 0.431 | 0.420 | 0.013 |
| TKEO Life satisfaction | 0.003 | 0.251 | 0.227 | 0.231 | 0.241 | 0.208 | 0.021 |
| PAC Life satisfaction | −0.034 | 0.375 | 0.325 | 0.366 | 0.364 | 0.349 | −0.014 |
| Auto. Life satisfaction | 0.054 | −0.007 | 0.029 | −0.080 | −0.061 | −0.079 | 0.174 |
| Average sleep | 0.138 | 0.001 | 0.028 | 0.036 | 0.023 | 0.030 | −0.065 |
| Time spent alone | −0.239 | 0.033 | −0.026 | 0.002 | 0.015 | 0.009 | 0.064 |
| Time spent with family | 0.072 | 0.001 | 0.030 | 0.008 | 0.004 | 0.001 | −0.012 |
| Time spent with friends | 0.064 | 0.100 | 0.113 | 0.105 | 0.091 | 0.106 | −0.025 |
| Time spent at work/studying | 0.040 | −0.033 | −0.027 | −0.029 | −0.031 | −0.023 | −0.005 |
| Time spent exercising | 0.092 | −0.018 | 0.016 | −0.015 | −0.017 | −0.017 | −0.006 |

**Table S7** Effect sizes (Pearson’s *r*) of the correlation between different outcomes and measures of affect. Outcomes correspond to demographic variables, variables estimating the propensity of individuals to perform an activity or being in the presence of others or measures of life satisfaction, meaning, and sleep. The correlations were estimated using our full sample of 7016 individuals each providing a minimum of 50 affect reports.
